# TRPML1 links lysosomal calcium to autophagosome biogenesis through the activation of the CaMKKβ/VPS34 pathway

**DOI:** 10.1038/s41467-019-13572-w

**Published:** 2019-12-10

**Authors:** A. Scotto Rosato, S. Montefusco, C. Soldati, S. Di Paola, A. Capuozzo, J. Monfregola, E. Polishchuk, A. Amabile, C. Grimm, A. Lombardo, M. A. De Matteis, A. Ballabio, D. L. Medina

**Affiliations:** 1 Telethon Institute of Genetics and Medicine (TIGEM), Pozzuoli, Naples, Italy; 2 Faculty of Medicine, Walther Straub Institute of Pharmacology and Toxicology, Ludwig-Maximilians-Universität, Munich, Germany; 3 Telethon Institute for Gene Therapy (SR-Tiget), Division of Regenerative Medicine, Stem Cells, and Gene Therapy, IRCCS San Raffaele Scientific Institute, Milan, Italy; 4 Vita-Salute San Raffaele University, 20132 Milan, Italy; 5 Department of Molecular Medicine and Medical Biotechnology, Federico II University, Naples, Italy; 6 Medical Genetics Unit, Department of Medical and Translational Science, Federico II University, Naples, Italy; 7 Baylor College of Medicine, Houston, Texas USA; 8 Jan and Dan Duncan Neurological Research Institute, Texas Children’s Hospital, Houston, Texas USA

**Keywords:** Macroautophagy, Calcium signalling, Ion channel signalling, Lysosomes

## Abstract

The lysosomal calcium channel TRPML1, whose mutations cause the lysosomal storage disorder (LSD) mucolipidosis type IV (MLIV), contributes to upregulate autophagic genes by inducing the nuclear translocation of the transcription factor EB (TFEB). Here we show that TRPML1 activation also induces autophagic vesicle (AV) biogenesis through the generation of phosphatidylinositol 3-phosphate (PI3P) and the recruitment of essential PI3P-binding proteins to the nascent phagophore in a TFEB-independent manner. Thus, TRPML1 activation of phagophore formation requires the calcium-dependent kinase CaMKKβ and AMPK, which increase the activation of ULK1 and VPS34 autophagic protein complexes. Consistently, cells from MLIV patients show a reduced recruitment of PI3P-binding proteins to the phagophore during autophagy induction, suggesting that altered AV biogenesis is part of the pathological features of this disease. Together, we show that TRPML1 is a multistep regulator of autophagy that may be targeted for therapeutic purposes to treat LSDs and other autophagic disorders.

## Abstract (web summary)

It was known that prolonged TRMPL1 activation induces TFEB translocation and upregulates autophagic gene regulation. Here, the authors show that acute TRMPL1 activation also induces autophagy through VPS34 and by lysosomal calcium release independent of TFEB.

## Introduction

Calcium is a universal second messenger that plays fundamental roles in cellular physiology. To allow rapid mobilization upon specific stimuli, calcium is kept at extremely low levels in the cytoplasm by its compartmentalization in organelles during resting conditions^1^. Endoplasmic reticulum (ER) and mitochondria^2^ are recognized as major cellular calcium stores; however, recent evidence indicates that lysosomal calcium also plays an important role in a variety of cellular processes^3^. Furthermore, impairment of lysosomal calcium homeostasis has been implicated in several human diseases such as lysosomal storage disorders (LSDs)^4–6^, neurodegenerative diseases^7–11^, muscular dystrophy, and cancer^12–14^. The non-selective cation channel TRPML1 is a major calcium-release channel on the lysosomal membrane^3^. TRPML1 activity is involved in a variety of membrane-trafficking processes such as lysosome to *trans*-Golgi-network retrograde trafficking, autophagic vesicle (AV)–lysosome fusion, lysosome reformation, and lysosomal exocytosis^3^. Mutations in *TRPML1* cause mucolipidosis type IV (MLIV: OMIM 252650), an autosomal recessive LSD characterized by psychomotor alterations, corneal opacities, and achlorhydria^15–17^. Cells from MLIV patients present defects in macroautophagy that are characterized by the accumulation of autophagic markers such as LC3 and p62^18–20^. Although the autophagic defects in MLIV, as well as in other LSDs, have been interpreted as the consequence of a global lysosomal dysfunction^21^, more specific mechanisms have not been identified.

Recent studies suggest that TRPML1 also plays a major role in lysosomal signaling during nutrient deprivation. Lysosomal calcium release through TRPML1 promotes the dephosphorylation of TFEB by the phosphatase calcineurin, thus inducing TFEB nuclear translocation and the consequent transcriptional activation of lysosomal and autophagic genes^22,23^. Thus, in addition to mediating the fusion of autophagosomes with lysosomes^19,24,25^, TRPML1 regulates autophagy by controlling the activity of the master transcriptional regulator of autophagy TFEB. Interestingly, TRPML1 and TFEB are involved in a feedback loop by which TRPML1 is at the same time a controller of TFEB activity and a downstream transcriptional target of TFEB and major effector of TFEB biological activity^23,26^. Here, by using genetic and pharmacological approaches to modulate TRPML1 activity, we show that TRPML1 can  regulate autophagy by an additional mechanism, which is not transcriptional and is independent of TFEB. Thus, TRPML1 can rapidly induce AV biogenesis through a signaling pathway that involves the activation of calcium/calmodulin-dependent protein kinase kinase β (CaMKKβ) and AMP-activated protein kinase (AMPK), the induction of the Beclin1/VPS34 autophagic complex, and the generation of phosphatidylinositol 3-phosphate (PI3P). This mechanism is pathophysiologically relevant, as MLIV patient cells show a reduced recruitment of PI3P-binding proteins to the phagophore during autophagy induction. Thus, our data identify TRPML1 as a multistep regulator of autophagy and a global controller of cell metabolism.

## Results

### TRPML1 induces AV formation independently of TFEB

**Fig. 1:**
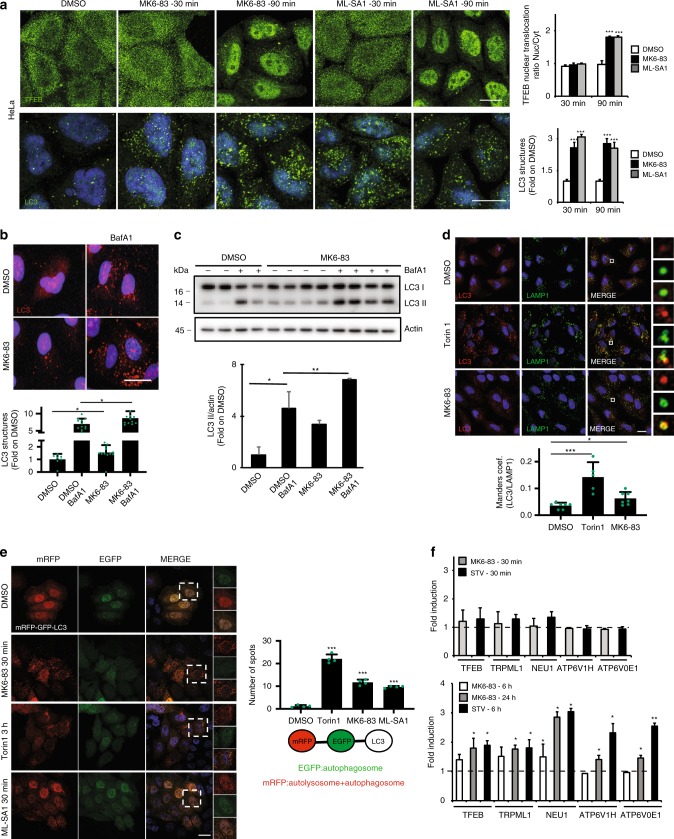
Agonist-mediated activation of TRPML1 can induces autophagy in a TFEB-independent manner. **a** Representative confocal images of endogenous TFEB and LC3 localization in HeLa cells treated with DMSO, MK6-83, or ML-SA1 at different time points (30–90 min). The plot represents the TFEB nuclear to cytosol ratio and the quantification of the LC3 puncta per cell. Values are means ± SD of *n* = 100 cells pooled from three independent experiments. **b** Representative confocal images of endogenous LC3 in HeLa cells upon DMSO or MK6-83 treatment alone or in the presence of the lysosomal inhibitor bafilomycin A1 (BafA1). The plot shows LC3 puncta fold induction to DMSO-treated cells. Values are means ± SD of *n* = 683 cells pooled from three independent experiments. **c** Representative image of immunoblot analysis of endogenous LC3 (LC3I-II) upon MK6-83 treatment alone or in the presence of BafA1. Plot shows the densitometry of LC3II band normalized to Actin. The data in the graphs on the right are mean values ± SD, *n* = 4 lysates per condition pooled from four independent experiments. **d** Representative confocal images of endogenous LAMP1 and LC3 upon MK6-83, Torin1, or DMSO treatment in HeLa cells. Plot shows the Manders coefficient of LC3-LAMP1 colocalization. Values are means ± SD of *n* = 215 cells pooled from three independent experiments. **e** Representative images from high-content assay of HeLa cells stably overexpressing mRFP-EGFP-LC3 plasmid treated with DMSO, Torin1, MK6-83, or ML-SA1. Plot shows the quantification of the autolysosome number (spots negative for GFP and positive for RFP). **f** Quantitative PCR showing the mRNA levels of a subset of TFEB target genes upon MK6-83 treatment at different time points (30 min, 6 and 24 h) and in control cells treated with HBSS. The data in the graphs are mean values ± SD, *n* = 3 samples per condition. Scale bar: 20 µm. *P*-values calculated by two-tailed Student’s *t*-test. *p-value < 0.05; **p-value < 0.01; ***p-value < 0.001.

We have recently shown that TRPML1 activity induces TFEB nuclear translocation through the activation of the phosphatase calcineurin and consequent dephosphorylation of TFEB during starvation^23^. This ability of TRPML1 to activate TFEB results in an enhanced expression of lysosomal and autophagic genes, and induction of autophagy. Consistently, silencing of TFEB reduces the effect of TRPML1 on autophagy induction^23^. However, the production of a functional protein from gene transcription to its translation can take significantly more time than calcium mobilization^1,27^. Thus, we asked whether the acute activation of TRPML1 could also contribute to the regulation of the autophagic pathway in a transcription-independent manner. Therefore, we analyzed critical steps of the autophagic pathway at several time points after pharmacological induction of TRPML1 channel activity using two synthetic agonists, MK6-83 and ML-SA1^5,28,29^. We found that both agonists increase LC3 puncta formation at all time points tested, 30 and 90 min (Fig. 1a). Also, we found that MK6-83-mediated elevation of LC3 puncta formation was further increased in cells overexpressing TRPML1 (Supplementary Fig. 1a). However, as MK6-83 is not TRPML1 selective^5,28,29^, we investigated its selectivity by depleting each of the three channels belonging to the TRPMLʼs family. We found that MK6-83 activity was fully inhibited in cells depleted of TRPML1, by using both genome editing or acute silencing, but not in cells depleted of TRPML2 or TRPML3, indicating that MK6-83 can induce AV formation through TRPML1 independently of the other channels (Supplementary Fig. 1b–e). In contrast to the more ubiquitous expression of TRPML1, the expression and subcellular localization of the other members of this family is tissue-specific and not restricted to the lysosomal compartment^20^. By using expression vectors carrying either *TRPML2* or *TRPML3*, we found that *TRPML3* overexpression, but not *TRPML2*, was able to increase LC3 puncta formation (Supplementary Fig. 1f). Similar results were obtained by testing the ability of synthetic agonist of either TRPML2 or TRPML3, ML2-SA1, and SN-2^30,31^, respectively. In accordance with our *TRPML2* overexpression data, ML2-SA1 was not able to induce LC3 puncta formation (Supplementary Fig. 1g). Conversely, SN-2 was able to weakly induce LC3 puncta formation in both wild-type (WT) and TRPML1-depleted cells (Supplementary Figs. 1g and 2a, b), indicating that TRPML3 may regulate autophagy independently of other TRPML members, most likely in tissues where it is highly expressed^20^.

Focusing on TRPML1, we also observed a further elevation of endogenous LC3 lipidation in the presence of MK6-83 and the lysosomal inhibitor bafilomycin A1, suggesting that TRPML1 activation induces AV maturation and autophagy activation (Fig. 1b, c). Similarly, overexpression of *TRPML1* increased LC3II lipidation and this effect was enhanced by bafilomycin A1 treatment (Supplementary Fig. 2c). Conversely, cells co-treated with MK6-83 and the TRPML1 inhibitor ML-SI3^32^ showed a reduction in LC3II formation compared with MK6-83 treatment alone (Supplementary Fig. 2d). Consistently, we found that both pharmacological activation of TRPML1 and *TRPML1* overexpression increased the AV–lysosome fusion as measured by the colocalization of LC3 with the lysosomal membrane protein LAMP1 (Fig. 1d and Supplementary Fig. 2e), an effect that was suppressed, at least partially, by the inhibitor of AV–lysosomal fusion vinblastine as well as by the silencing of the autophagic SNARE protein syntaxin-17^33,34^ (Supplementary Fig. 3a–c). Notably, we also found some LAMP1-positive vesicles stained with LC3 even after AV–lysosomal fusion inhibition, suggesting that TRPML1 activation may also induce lipidation of other structures, most likely endolysosomal vesicles, a phenomenon that has been previously observed in cells treated with other autophagy modulators^35^.

Electron microscopy analysis of a stable U2OS cell line overexpressing LC3-GFP also showed an increase in the number of autolysosomes upon MK6-83 treatment (Supplementary Fig. 4a). Similarly, an increase in the number of autolysosomes (enhanced green fluorescent protein (EGFP)-negative and red fluorescent protein (RFP)-positive) was observed in a stable HeLa cell line overexpressing the autophagic reporter RFP-EGFP-LC3^33^ (Fig. 1e). Together, these data indicate that activation of TRPML1 induces autophagosome formation and facilitates AV–lysosomal fusion.

To investigate the mechanism by which TRPML1 induces autophagosome formation, we examined the subcellular localization of TFEB, as TRPML1 was shown to induce TFEB nuclear translocation^23^. Notably, 30 min after TRPML1 induction, a time when we observed a significant increase in the number of autophagosomes, TFEB was found in the cytoplasm (Fig. 1a) and the mRNA levels of a set of TFEB target genes were not increased (Fig. 1f), whereas at later time points of TRPML1 activation TFEB was detected in the nucleus (Fig. 1a) and the same set of mRNAs were upregulated (Fig. 1f). Similarly, co-treatment of MK6-83 with the transcription inhibitor actinomycin D (ACT-D) still induced LC3 puncta formation, excluding a transcriptional effect during acute treatment (Supplementary Fig. 4b, c). Furthermore, acute MK6-83 treatment was able to induce LC3 puncta formation both in cells depleted of TFEB by small interfering RNAs (siRNAs) and, to a lesser extent, in *TFEB/TFE3* double knockout (KO) cells^36^ (Supplementary Fig. 4d–f). In agreement with recent work^37^, the activation of TRPML1 by MK6-83 in normal nutrient conditions did not affect the phosphorylation of the mTORC1 substrates T389-PS6K and S6 ribosomal protein (Supplementary Fig. 4g, h), thus excluding a role of mammalian target of rapamycin (mTOR).

Together, these data indicate that acute TRPML1 activation is sufficient to induce autophagosome biogenesis in a TFEB-independent manner, suggesting that these effects are mediated by a different mechanism.

### Activation of TRPML1 recruits PI3P-binding proteins to AVs

**Fig. 2:**
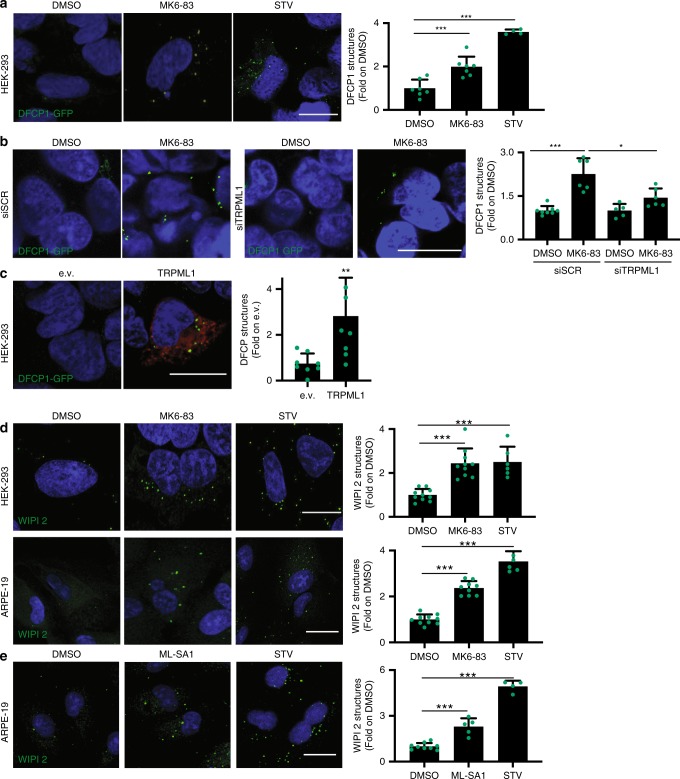
TRPML1 activation increases the recruitment of the PI3P-binding proteins DFCP1 and WIPI2. **a** Representative confocal images of HEK-293 cells stably overexpressing DFCP1-GFP treated with DMSO, HBSS, or MK6-83. The plot shows the quantification of DFCP1 puncta formation as fold induction to DMSO-treated cells. Values are means ± SD of *n* > 1000 cells pooled from three independent experiments. **b** Representative confocal images of HEK-293 cells stably expressing DFCP1-GFP treated with a single siRNA molecule targeting TRPML1 (siTRPML1) or a scramble sequence (SCR) followed by treatment with MK6-83, DMSO, or HBSS. The plot shows the quantification of DFCP1 puncta formation as fold induction to DMSO-treated cells. Values are means ± SD of *n* > 1000 cells pooled from three independent experiments. **c** HEK-293 stably expressing DFCP1-GFP were transiently transfected with a vector encoding TRPML1 tagged with FLAG (in red) or an empty vector (e.v.). The plot shows the quantification of DFCP1 puncta as fold induction to the e.v.-transfected cells. Values are means ± SD of *n* = 733 cells pooled from three independent experiments. **d** Representative confocal images of endogenous WIPI2 in HEK-293 cells and in ARPE-19 cells upon treatment with MK6-83, DMSO, or STV (HBSS). Plots show the WIPI2 puncta quantification as fold induction to DMSO-treated cells. Values are means ± SD of *n* = 167 cells pooled from three independent experiments (HEK-293) and *n* = 320 cells pooled from two independent experiments (ARPE-19). **e** Representative confocal images of ARPE-19 cells treated with ML-SA1, DMSO, or STV (HBSS). Plot shows WIPI2 quantification as fold induction to DMSO-treated cells. Values are means ± SD of *n* = 722 cells pooled from three independent experiments. *P*-values calculated by two tails Student’s *t*-test. Scale bar: 20 µm. *p-value < 0.05; **p-value < 0.01; ***p-value < 0.001.

To identify the mechanism by which acute TRPML1 activation induces AV formation, we investigated the potential role of this lysosomal calcium channel in autophagy initiation. In mammalian cells, PI3P-enriched ER subdomains, known as omegasomes, act as platforms for AV formation upon autophagy induction^38^. To test whether TRPML1 is involved in phagophore formation, we acutely stimulated TRPML1 in HEK-293 cells that stably overexpress the omegasome marker double FYVE-containing protein 1 (DFCP1) fused to GFP (DFCP1-GFP)^38^. Cells treated with MK6-83 for 30 min showed a significant increase in the formation of DFCP1-GFP-puncta compared with vehicle-treated cells (Fig. 2a). The knockdown of *TRPML1* decreased the induction of DFCP1-GFP puncta formation upon MK6-83 treatment confirming the selectivity of the agonist (Fig. 2b). Consistent with the results obtained with the TRPML1 activator MK6-83, overexpression of *TRPML1* increased DFCP1-GFP puncta compared with the control vector (Fig. 2c). Moreover, we found that MK6-83-mediated activation (30 min) of TRPML1 induced the recruitment of endogenous WD-repeat domain phosphoinositide-interacting  WIPI2  puncta, an essential effector of the nascent AV^39^, in both HEK-293 and ARPE-19 cells (Fig. 2d). Similar results were obtained in ARPE-19 cells treated with ML-SA1 (Fig. 2e). WIPI2 is recruited to early PI3P-enriched autophagosomal structures along with ATG16L1 and is required for LC3 lipidation^40^. We found that upon TRPML1 activation, WIPI2 puncta colocalize with the endogenous ATG16L1 protein (Supplementary Fig. 5a), indicating that TRPML1 activation induces the formation of nascent phagophores that recruit the machinery of LC3 lipidation. Together, these data suggest that the direct activation of TRPML1 increases the recruitment of essential PI3P-binding proteins to the nascent AVs.

### TRPML1-mediated generation of PI3P requires VPS34 and ULK1

**Fig. 3:**
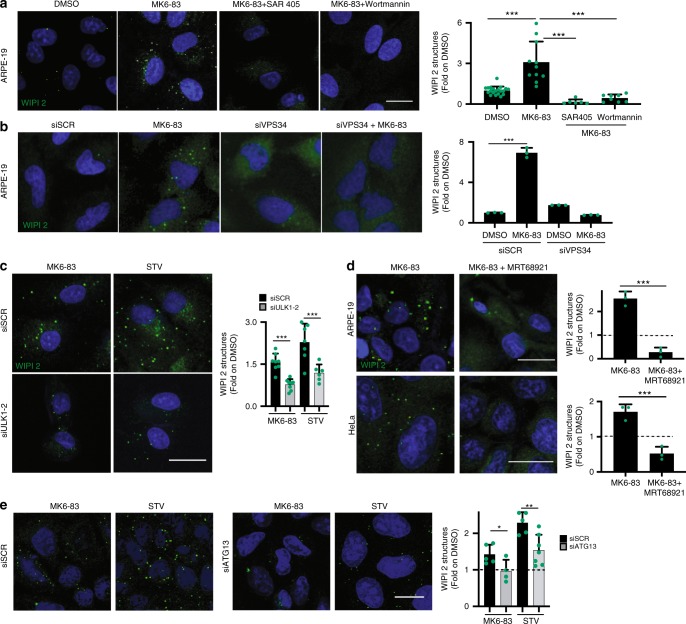
PI3P generation through TRPML1 requires both hVPS34 and ULK1. **a** Representative confocal images of endogenous WIPI2 in ARPE-19 cells treated with DMSO and MK6-83 alone or in combination with SAR405 or wortmannin. Plot shows the quantification of WIPI2 puncta as fold induction to DMSO-treated cells. Values are means ± SD of *n* > 1000 cells pooled from three independent experiments. **b** Representative images from high-content assay of endogenous WIPI2 in ARPE-19 cells treated with a pool of three siRNA targeting VPS34 (siVPS34) or a scramble sequence. Values are means ± SD of *n* = 200 cells pooled from three independent experiments. **c** Representative images from cell-based high-content imaging assay of endogenous WIPI2 in ARPE-19 cells silenced with siRNA molecules targeting ULK1 and ULK2, followed by treatment with MK6-83 or STV (HBSS). Plot shows the quantification of WIPI2 puncta as fold induction to DMSO-treated cells. Values are means ± SD of *n* > 1000 cells pooled from three independent experiments. **d** Representative confocal images of endogenous WIPI2 in ARPE-19 and HeLa cells treated with MK6-83 alone or in combination with the ULK1 inhibitor (MRT-68921). Plots show the quantification of WIPI2 puncta as fold to DMSO. Values are means ± SD of *n* > 1000 cells pooled from four independent experiments. **e** Representative confocal images of endogenous WIPI2 in HeLa cells silenced with a pool of three siRNA molecules targeting ATG13 (siATG13) followed by treatment with MK6-83 or STV (HBSS). Plot shows the quantification of WIPI2 puncta as fold induction to DMSO-treated cells. Values are means ± SD of *n* > 1000 cells pooled from three independent experiments. *P*-values calculated by two tails Student’s *t*-test. Scale bar: 20 µm. *p-value < 0.05; **p-value < 0.01; ***p-value < 0.001.

The recruitment of DFCP1 and WIPI2 to the nascent AV requires the generation of PI3P by the major autophagic protein complex hVPS34/class III phosphoinositide-3-kinase complex 1 (PI3KC3-C1)^41^. We found that specific PI3K inhibitors, such as wortmannin and the more selective compound SAR405^42^, were able to fully block the effects of MK6-83-mediated activation of TRPML1 on WIPI2 puncta formation (30 min treatment; Fig. 3a). Similarly, siRNA-mediated silencing of *VPS34* significantly reduced WIPI2 puncta in cells treated with MK6-83 (Fig. 3b). In agreement with the role of WIPI2 in the recruitment of LC3 to the phagophore^40^, we also found that pharmacological inhibition of VPS34 with SAR405 significantly reduced MK6-83-mediated induction of LC3 lipidation and LC3 puncta formation, as measured by immunoblot and immunofluorescence, respectively (Supplementary Fig. 5b, c). Thus, these results further suggest that TRPML1 can promote the formation and maturation of AVs.

Furthermore, both MK6-83 and ML-SA1 agonists were able to increase the production of PI3P, which was measured using a highly specific fluorescently labeled PX domain probe^43,44^, in a VPS34-dependent manner (Supplementary Fig. 5d). Most importantly, we also observed an elevation of immunoprecipitated VPS34 lipid kinase activity upon MK6-83 and ML-SA1 treatments (Supplementary Fig. 5e). The induction of autophagy requires the activation of ULK1 protein kinase complex, an event that is upstream of the activation of the VPS34 complex^45^. Thus, we tested whether MK6-83-mediated induction of WIPI2 puncta formation could be affected by the depletion of ULK1 complex activity. Silencing of *ULK1/2* fully inhibited WIPI2 puncta formation upon MK6-83 treatment (Fig. 3c and Supplementary Fig. 6a, b, j, l). Similar results were obtained using an inhibitor of ULK1 (MRT-68921) in two different cell lines (Fig. 3d). Finally, the ability of MK6-83 to induce WIPI2 puncta was significantly reduced in cells depleted of *ATG13* (Fig. 3e and Supplementary Fig. 6e, l) and in FIP200 KO mouse embryonic fibroblasts (MEFs) (Supplementary Fig. 7a), two critical components of the ULK1 complex, which are required for its assembly and for full ULK1 protein kinase activity^46–49^. Collectively, these results strongly indicate that activation of TRPML1 is sufficient to promote the generation of PI3P and AV biogenesis through activation of the two major protein complexes involved in autophagy initiation, ULK1, and VPS34.

### TRPML1-mediated induction of AV formation requires CaMKKβ

**Fig. 4:**
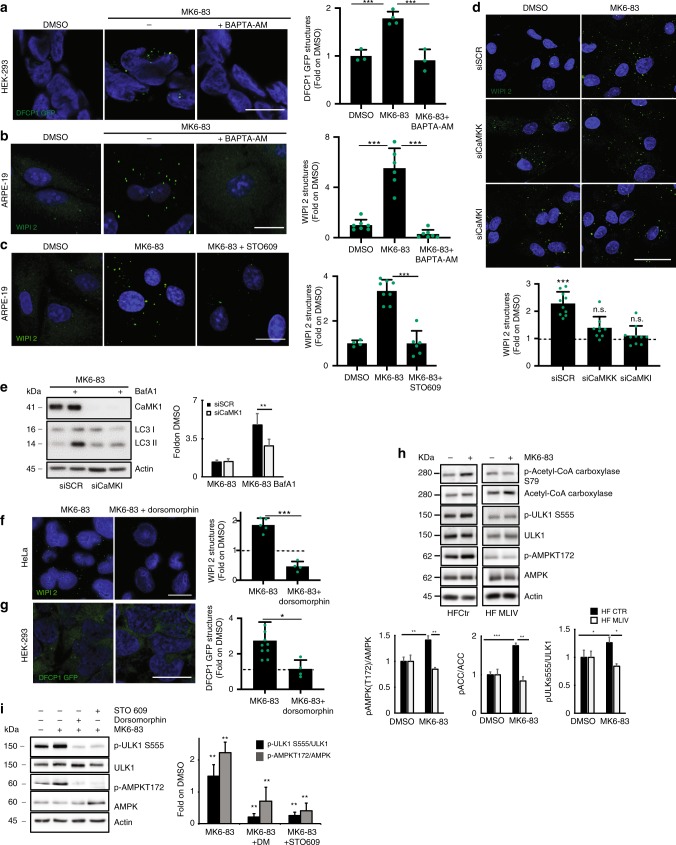
TRPML1-mediated lysosomal Ca2+ release induces AV biogenesis through CaMKKβ. **a** Representative images of HEK-293 overexpressing DFCP1-GFP treated with DMSO and MK6-83 ± BAPTA-AM. **b** Representative images of WIPI2 in ARPE-19 treated with DMSO and MK6-83 ± BAPTA-AM. **c** Representative images of WIPI2 in ARPE-19 treated with DMSO and MK6-83 alone or in co-treatment with STO-609. **d** Representative images of WIPI2 in ARPE-19 treated with a pool of three siRNAs targeting CaMKKβ or CaMKI ± MK6-83. **e** Representative image of immunoblot analysis of LC3 (LC3I-II) in HeLa cells silenced with siRNAs targeting CaMKI and treated with MK6-83 ± bafilomycin A1 (BafA1). Plot shows the densitometry of LC3II normalized to actin as fold induction to DMSO. The data in the graphs on the right are mean values ± SD, *n* = 4 lysates per condition pooled from four independent experiments. **f** Representative images of WIPI2 in ARPE-19 treated with DMSO and MK6-83 ± dorsomorphin. **g** Representative images of HEK-293 overexpressing DFCP1-GFP treated with DMSO and MK6-83 ± dorsomorphin (DM). **h** Representative image of immunoblot analysis of phosphorylation AMPK on T172 and ULK1 on S555, Acetyl-CoA Carboxylase (ACC) on S79, upon MK6-83 treatment. Plot shows the densitometry of p-AMPK T172, p-ULK1 S555, and p-ACC S79 on AMPK, ULK1, and ACC normalized on Actin as fold induction to DMSO. The data in the graphs are mean values ± SD, *n* = 3 lysates per condition pooled from three independent experiments. **i** Representative image of immunoblot analysis of phosphorylation of AMPK on T172 and its substrate ULK1 on S555 upon MK6-83 treatment ± STO-609 or dorsomorphin. Plot shows the densitometry of p-AMPK T172 and p-ULK1 S555 on AMPK and ULK1 normalized on actin as fold induction to DMSO. The data in the graphs are mean values ± SD, *n* = 4 lysates pooled from four independent experiments. **a**–**d**, **f**, **g** Plot shows the quantification of -WIPI2 (**b**–**d**, **f**), -DFCP (**a**, **g**) positive puncta as fold induction to DMSO-treated cells. Values are means ± SD of *n* = 208 (**a**), *n* = 225 (**b**), *n* = 1169 (**c**), *n* = 610 (**d**), *n* = 385 (**f**), *n* = 687 (**g**) cells pooled from three independent experiments. Scale bar: 20 µm. *p-value < 0.05; **p-value < 0.01; ***p-value < 0.001.

Intrigued by the link between TRPML1 activation and AV biogenesis, we investigated the molecular mechanisms activated downstream of the lysosomal calcium signaling. First, we asked whether TRPML1-mediated lysosomal calcium release is required for the recruitment of the autophagic PI3P-binding proteins to the nascent AV. To this purpose, we treated ARPE-19 and DFCP1-GFP HEK-293 cells with MK6-83 alone or in combination with the intracellular fast Ca^2+^ chelator BAPTA-AM^23^. We observed that BAPTA-AM fully abolished the elevation of DFCP1 and WIPI2 puncta induced by MK6-83 (Fig. 4a, b), strongly suggesting that calcium is involved in agonist-mediated TRPML1 effects on AV biogenesis. Interestingly, co-treatment of MK6-83 with the slow Ca^2+^ chelator EGTA-AM^23^ did not affect WIPI2 puncta formation, suggesting that local calcium release is involved in TRPML1-mediated induction of AV formation (Supplementary Fig. 7b). Most importantly, although the overexpression of GFP-tagged TRPML1, localizing in the lysosome, can induce WIPI2 puncta, the overexpression of a non-conducting pore mutant version of TRPML1 (TRPML1-DDKK^50^) does not (Supplementary Fig. 7c), confirming that the channel activity is required for phagophore formation.

The CaMKKβ is known to play a major role in the effects of intracellular Ca^2+^ on autophagy induction^51^. We found that the use of the CaMKKβ inhibitor STO-609^51^ significantly reduced the formation of WIPI2 puncta in cells treated with MK6-83 (Fig. 4c). Consistently, silencing of *CaMKKβ* (Supplementary Fig. 6a, b, i, l) and its downstream effector calcium/calmodulin-dependent protein kinase I (CaMKI) (Supplementary Fig. 6a, b, i, l) significantly reduced WIPI2 puncta formation upon direct activation of TRPML1 with MK6-83 (Fig. 4d). Moreover, the depletion of *CaMKI* caused a reduction of LC3 lipidation upon MK6-83 treatment, as revealed by the co-treatment with bafilomycin A1 (Fig. 4e). The silencing of *CaMKKβ* or *CaMKI* did not affect TRPML1-mediated induction of TFEB nuclear translocation, indicating that these kinases are involved in TRPML1-dependent AV biogenesis but not in TRPML1-dependent TFEB activation (Supplementary Fig. 7d). Thus, these results suggest the functional involvement of CaMKKβ/CaMKI in regulating WIPI2 recruitment to the forming AV upon TRPML1 activation. Intracellular calcium levels can trigger autophagy by a CaMKKβ-mediated activation of AMPK^51–53^. We observed that the AMPK inhibitor dorsomorphin (DM)^51^ resulted in a significant reduction of MK6-83-induced WIPI2 and DFCP1-GFP puncta formation, in HeLa cells and HEK-293 cells, respectively (Fig. 4f, g). Moreover, the depletion of *AMPK* by siRNAs (Supplementary Fig. 6a, b, l) significantly inhibits MK6-83-induced WIPI2 puncta formation. Also, we found that MK6-83 was able to promote the activation of AMPK by elevating its phosphorylation on threonine 172 in WT fibroblasts but not in MLIV patient cells (Fig. 4h). Interestingly, AMPK induces autophagy by directly activating ULK1 via its phosphorylation on serine 555^54,55^. Consistently, MK6-83 treatment also enhances ULK1 S555 phosphorylation^56^. Conversely, co-treatment with STO-609 or DM completely abolished MK6-83-mediated activation of AMPK/ULK1 (Fig. 4i). In agreement with this result, overexpression of *TRPML1* was able to enhance phosphorylation of ULK1 on serine 555 and Acetyl-CoA Carboxylase (ACC) on serine 79, both well-known direct AMPK substrates^57^ (Supplementary Fig. 7f). Interestingly, treatment with SN-2 in *TRPML3*-overexpressing cells was able to induce the phosphorylation of the AMPK substrates ACC and ULK1, suggesting that in cells where TRPML3 is physiologically expressed at higher levels it may activate AV formation similar to TRPML1 (Supplementary Fig. 7g). Together, these results indicate that the induction of lysosomal TRPML1 activates the CaMKKβ/AMPK pathway to promote AV biogenesis through ULK1 activation.

### TRPML1 activation promotes Beclin1 S15 phosphorylation

**Fig. 5:**
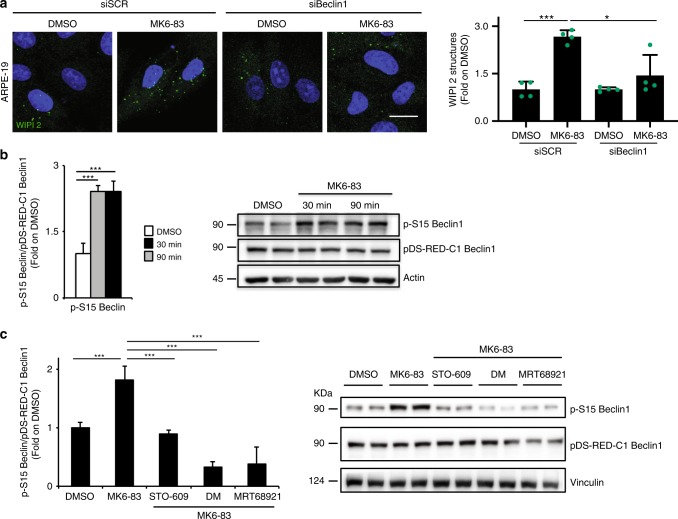
Pharmacological activation of TRPML1 enhances Beclin1 S15 phosphorylation through the CaMKK/AMPK/ULK signaling pathway. **a** Representative confocal images of endogenous WIPI2 in ARPE-19 cells treated with with a pool of three siRNA molecules targeting Beclin1 (siBeclin) followed by treatment with MK6-83. Plot shows the quantification of WIPI2-positive puncta as fold induction to DMSO-treated cells. Values are means ± SD of *n* = 616 cells pooled from three independent experiments. **b** Representative image of immunoblot analysis of phosphorylation of pDS-RED-C1-Beclin1 on serine 15 upon MK6-83 treatment at 30 and 90 min. Plot shows the densitometry of p-Beclin1 S15 on pDS-RED-C1-Beclin1 normalized on Vinculin as fold induction to DMSO-treated cells. The data in the graphs on the left are mean values ± SD, *n* = 6 lysates per condition pooled from three independent experiments. **c** Representative image of immunoblot analysis of phosphorylation of pDS-RED-C1-Beclin1 on serine 15 upon MK6-83 treatment alone or in combination with STO-609, dorsomorphin (DM), or MRT-68921. The data in the graphs on the left are mean values ± SD, *n* = 6 lysates per condition pooled from three independent experiments. *P*-values calculated by two tails Student’s *t*-test. Scale bar: 20 µm. *p-value < 0.05; **p-value < 0.01; ***p-value < 0.001.

The autophagy protein Beclin1 is a central regulator of autophagy and functions through its interaction with the Class III PI3K VPS34, VPS15, and the autophagy protein ATG14 in the initial stages of AV formation^58^. We asked whether Beclin1 is necessary for TRPML1-mediated induction of AV formation. Interestingly, we found that the depletion of *Beclin1* by siRNA-mediated silencing can significantly reduce TRPML1-mediated induction of WIPI2 spots upon MK6-83 treatment (Fig. 5a and Supplementary Fig. 6g, l, m).

Phosphorylation of Beclin1 on a specific serine (S15 in humans) by ULK1 is required for full autophagy induction through the activation of ATG14L-containing VPS34 complexes^59^. We found that MK6-83-mediated activation of TRPML1 induces the phosphorylation of Beclin1 S15 as detected by using phospho-specific antibodies that recognize this phosphoserine in cells overexpressing WT pDS-RED-C1-Beclin1 (Fig. 5b). Next, we sought to test whether the MK6-83-mediated induction of the CaMKKβ/AMPK/ULK1 pathway mediates the phosphorylation of Beclin1 on S15. Therefore, *Beclin1*-overexpressing cells were stimulated with MK6-83 alone or in combination with inhibitors of CaMKKβ (STO-609), AMPK (DM), or ULK1 (MRT-68921). By following Beclin1 phosphorylation on S15, we found that these inhibitors were able to reduce the phosphorylation of Beclin1 (Fig. 5c), suggesting that MK6-83 can enhance VPS34 activity through the phosphorylation of Beclin1 on S15 via the CaMKKβ/AMPK/ULK1 pathway.

### WIPI2 recruitment is reduced in cells from MLIV patients

**Fig. 6:**
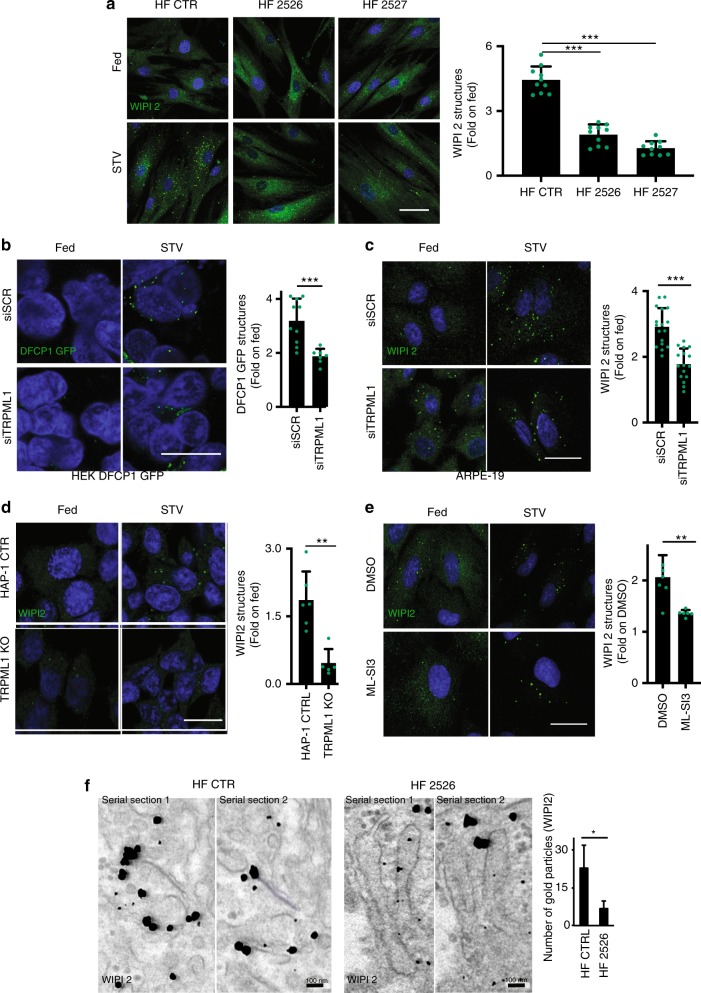
Induction of AV formation is reduced in Mucolipidosis type IV cells. **a** Representative confocal images of endogenous WIPI2 in human fibroblasts derived from a healthy individual (HF CTR) or from mucolipidosis type IV patient cell lines (GM02527, GM02526) incubated in complete medium or STV (HBSS). Plot shows the number of WIPI2 puncta per cell. Values are means ± SD of *n* = 400 total cells from three independent experiments. **b** Representative confocal images of HEK-293 cells overexpressing DFCP1-GFP treated with a single siRNA molecule targeting TRPML1 (siTRPML1) followed by treatment with complete medium (Fed) or STV (Starvation HBSS). Plot shows the quantification of DFCP1 positive puncta as fold induction to DMSO-treated cells. Values are means ± SD of *n* > 1000 cells pooled from three independent experiments. **c** Representative confocal images of endogenous WIPI2 in ARPE-19 cells treated with siRNA molecules targeting TRPML1 followed by incubation in complete medium or STV (Starvation HBSS). Plot shows the quantification of WIPI2-positive puncta as fold induction to DMSO-treated cells. Values are means ± SD of *n* = 391 cells pooled from three independent experiments. **d** Representative confocal images of endogenous WIPI2 in HAP-1 wild-type (CTR) or KO for TRPML1 incubated in complete medium or STV (HBSS). Plot shows the quantification of WIPI2 puncta as fold induction to DMSO-treated cells. Values are means ± SD of *n* = 925 cells pooled from three independent experiments. **e** Representative confocal images of endogenous WIPI2 in ARPE-19 cells, followed by treatment with the synthetic inhibitor of TRPML1 (ML-SI3). Plot shows the quantification of WIPI2 puncta as fold induction to DMSO-treated cells. Values are means ± SD of *n* = 286 cells pooled from three independent experiments. **f** TEM images of endogenous WIPI2 labeled by immunogold technique, in human fibroblasts derived from a healthy individual (HF CTRL) or derived from a mucolipidosis type IV patient (HF 2526). The plot shows the quantification of WIPI2 puncta. Values are means ± SD of *n* = 726 cells pooled from three independent experiments. *P*-values calculated by two-tailed Student’s *t*-test. Scale bar: 20 µm, **f**: 100 nm. *p-value < 0.05; **p-value < 0.01; ***p-value < 0.001.

*TRPML1* mutations cause the human lysosomal storage disease mucolipidosis IV^15,60^. We investigated whether the induction of PI3P-binding protein recruitment was impaired in human fibroblasts derived from different MLIV patients (GM02526 and GM02527). In nutrient deprivation conditions such as Hank’s balanced salt solution (HBSS) or amino acid starvation, WIPI2 puncta formation was significantly induced in WT fibroblasts but not in MLIV patient cells (Fig. 6a and Supplementary Fig. 8a). Similarly, starvation-mediated induction of WIPI2 puncta was reduced in ARPE-19 cells depleted of *TRPML1* by genome editing (Supplementary Fig. 8b). Conversely, overexpression of WT *TRPML1* was able to restore WIPI2 and autophagic induction in *TRPML1*-depleted cells (Supplementary Fig. 8c). A reduction of both DFCP1-GFP and WIPI2 puncta was observed by acute silencing of *TRPML1* in stably DFCP1-GFP-transfected HEK-293 cells and ARPE-19 (Fig. 6b, c). Indeed, the induction of WIPI2 puncta was reduced in a MLIV cellular model generated by genome editing in human HAP-1 cells (Fig. 6d). Similar reduction of both WIPI2 (Fig. 6e) and LC3 (Supplementary Fig. 8d) puncta formation during starvation was also observed 1 h after treatment with the antagonist of TRPML1, ML-SI3 (Supplementary Fig. 9a). The inhibitory effects of ML-SI3 during starvation was observed also in cells silenced for *TRPML3*, indicating that TRPML3 does not play a major role in WIPI2 puncta formation during starvation (Supplementary Fig. 9b). Conversely, the activation of TRPML1 with MK6-83 has an additive effect elevating the formation of WIPI2 puncta during starvation (Supplementary Fig. 9c). We also confirmed a reduction of endogenous WIPI2 decorating phagophore-like structures and associated ER membranes by immuno-electron microscopy analysis of starved human MLIV cells (Fig. 6f)^61^. Notably, the reduction of WIPI2 puncta formation observed in MLIV cells was not due to the down-regulation of WIPI2 mRNA or protein levels, as quantitative PCR (qPCR) or immunoblot analysis in normal nutrient and starvation conditions resulted in similar WIPI2 content in both MLIV and WT fibroblasts (Supplementary Fig. 10a, b). To further confirm that the reduction of PI3P-binding proteins in *TRPML1*-depleted cells was due to a decrease in PI3P generation, we measured PI3P using the PX domain reporter in *TRPML1*-KO ARPE-19 cell line generated by genome editing. Indeed, we found a significant reduction of PX-positive puncta in KO cells compared to WT cells during autophagy induction (Supplementary Fig. 10c).

Altogether, our observations reveal a defective generation of PI3P and an impaired recruitment of essential PI3P-binding proteins to the nascent AV in various models of MLIV, suggesting that this may be a relevant mechanism in the disease pathogenesis.

## Discussion

Mammalian TRPML1 is a ubiquitously expressed non-selective, cation-permeable channel^24^, which mediates the release of Ca^2+^ from lysosomal or late endosomal lumen into the cytosol. TRPML1 is indispensable in the processes of endocytosis, membrane trafficking, lysosomal exocytosis, and lysosomal biogenesis^23,28,29,62–65^. Consistent with its role in lysosomal biogenesis, mutations in the *TRPML1* gene cause the lysosomal storage disease MLIV, which is an autosomal recessive disorder characterized by delayed psychomotor development and vision impairment^15–17^. Notably, we showed that transcriptional activation of TRPML1 via TFEB promotes clearance of pathological storage in a variety of LSDs through the activation of lysosomal exocytosis^65^.

Experimental- and structural-based studies indicate that TRPML1 is regulated by pH, Ca^2+^, and phosphoinositides in a combined manner reflecting its ability to modulate highly dynamic trafficking processes such as endocytosis^66–69^. Interestingly, TRPML1 release activity can be modulated by nutrient deprivation activating a TFEB-driven transcriptional program through the induction of its nuclear translocation^23^. This mechanism may have an important role in the regulation of sustained autophagy during prolonged starvation conditions. However, although the complete process of transcription and subsequent translation of autophagic genes may require long kinetics, the acute activation of TRPML1-mediated lysosomal calcium mobilization may have consequences in the autophagy machinery at a shorter timescale. In the present study, we used synthetic agonists of TRPML1 to comprehensively investigate the effects of direct TRPML1 activation on autophagy. We found that acute pharmacological activation of TRPML1 rapidly induces AV formation and AV–lysosome fusion at early time points when TFEB is inactive being located predominantly in the cytosol. Our data are in agreement with previous work indicating that TRPML1 positively regulates membrane-trafficking processes such as vesicular fusion/fission events and autophagy^5,23,32,50,65,68,70–73^. There are also a few reports suggesting that TRPML1 modulation can block AV–lysosomal fusion^74,75^, although these observations might be explained by differences in the experimental conditions used and/or levels of *TRPML1* overexpression. From a therapeutic perspective, it would be interesting to investigate whether excessive *TRPML1* expression/activation may impair both lysosomal ionic homeostasis and function promoting deleterious effects in specific cell types (e.g., cancer cells).

**Fig. 7:**
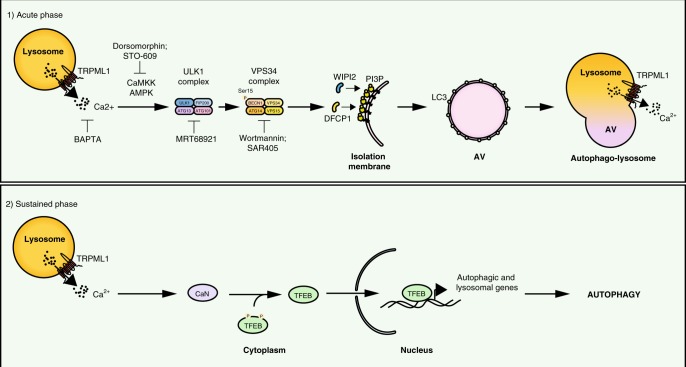
Model of TRPML1 regulation of autophagy. Model of multistep regulation of autophagy by TRPML1. TRPML1 modulates autophagy in two steps: (1) by rapidly inducing AV biogenesis and AV–lysosomal fusion through the activation of the calcium/calmodulin-dependent protein kinase kinase (CaMKKβ)-mediated signaling and the two major protein complexes involved in autophagy initiation, ULK1 and hVPS34, and (2) by inducing TFEB nuclear translocation that promotes the transcription of autophagic and lysosomal genes, which is necessary for a sustained induction of autophagy.

Our results indicate that acute TRPML1 activation induces AV formation independently of TFEB, suggesting that TRPML1 can modulate autophagy both in a rapid way, by promoting AV biogenesis and AV–lysosome fusion, and, in a sustained way, by inducing TFEB nuclear translocation and the transcription of autophagic and lysosomal genes, necessary to provide autophagic constituents during prolonged autophagy induction. Also, this mechanism is involved in a feedback loop by which TFEB positively regulates TRPML1 mRNA expression to further support long-term autophagy induction (working model Fig. 7)^76^.

In addition to TRPML1, the subfamily of TRPMLs includes TRPML2 and TRPML3. In contrast to TRPML1, these two channels show a very restricted tissue-specific expression and partially colocalize with late endosome/lysosomes^24,30,62,77^. By using cells depleted of each one of the channels, we found that TRPML1 activation is sufficient to induce AV biogenesis, also in the absence of *TRPML2* or *TRPML3*. Then, by overexpressing *TRPML2* and *TRPML3*, we confirmed that TRPML3, but not TRPML2, can induce LC3 puncta accumulation, as previously demonstrated^78–81^. Similarly, the selective agonist of TRPML3, SN-2, was able to weakly induce LC3 puncta formation in both WT and TRPML1-depleted cells, indicating that TRPML3 may also modulate autophagy independently of the other channels, although most likely in tissues where it is physiologically more expressed^20^. Although a few articles have described that heterologously expressed TRPML protein subunits can interact with each other^3,78,82^, evidences are suggesting that endogenous TRPML subunits only partially colocalize and therefore exist mostly as homomeric channels^82^. Our observations are in agreement with these evidences, although further studies are necessary to fully understand whether endogenous TRPML3 activation can replace TRPML1-mediated autophagy effects. The answer to this question might be relevant for the development of therapeutics using selective TRPML3 agonists to revert the human MLIV phenotype.

In mammals, the initiation of phagophore formation is regulated by the sequential activation of the ATG1/ULK complex and the autophagy-specific PI3KC3-C1 (or VPS34)^41,45^. PI3KC3-C1 kinase activity generates PI3P at the pre-autophagosomal structure called omegasome^38^. Membrane-bound PI3P is recognized by proteins such as DFCP1 and WIPI proteins^83^. Here we found that the activation of TRPML1 triggers the recruitment of PI3P-binding proteins DCFP1 and WIPI2, which are essential for nascent AVs. We also observed that upon TRPML1 activation, WIPI2-positive vesicles colocalize with ATG16L1, an essential component of the LC3 lipidation complex. WIPI2 is known to function downstream of PIK3C3/VPS34 and upstream of both the ATG12 and LC3 ubiquitin-like conjugation systems, and to trigger later steps of autophagosome generation^40^. Thus, our data suggest that TRPML1 activation and lysosomal calcium may also play a role in AV maturation through the induction of LC3 lipidation. Consistent with this hypothesis, we also found that TRPML1 activation increases AV–lysosomal fusion, a process that can be pharmacologically inhibited by vinblastine or by silencing of the autophagic SNARE syntaxin-17. Intriguingly, we observed that in addition to promoting autophagosome formation and autophagosome–lysosome fusion, TRPML1 activation also induces LC3 lipidation of endolysosomal structures, an effect that was previously observed in cells treated with other autophagy modulators^35^.

The recruitment of DFCP1 and WIPI2, upon TRPML1 activation, is likely a consequence of the elevation of PI3P levels, which requires both ULK1 and VPS34 activity, two essential protein complexes involved in the first steps of autophagy initiation^38^. Interestingly, we found defects in the induction of PI3P-binding protein recruitment to phagophore in fibroblasts from MLIV patients who carry loss-of-function mutations in the *TRPML1* gene. These results indicate that TRPML1 activity can activate the ULK1 complex and subsequently VPS34, and suggest that the impairment of this pathway is a relevant mechanism in MLIV pathogenesis.

The role of intracellular calcium levels during phagophore formation as well as the downstream pathways involved in this process have not been fully elucidated. Previous studies demonstrated that binding of PIK3C3/VPS34 to calmodulin and calcium is required for the activity of VPS34 during bacterial infection^84,85^. CaMKKβ can activate autophagy through both AMPK-dependent^51–53^ and -independent mechanisms^86^. Also, rapid AMPK activation during autophagy initiation was described during acute (<30 min) starvation. Interestingly, this acute response involves modulation of AMPK/ULK1 complexes, whereas it does not affect the more abundant ULK1/ATG13 complex^87^. Whether lysosomal calcium can promote autophagosome biogenesis through these pathways is completely unknown. Here we found that TRPML1-mediated signaling requires the activation of CaMKKβ and its substrate AMPK, a major nutrient-sensing kinase that regulates autophagy through phosphorylation of both ULK1 and Beclin1, and the subsequent induction of the VPS34 complex^54,88,89^. Thus, although acute TRPML1 activation may induce AV formation through CaMKKβ/AMPK pathway, prolonged starvation can induce autophagy through TFEB. Interestingly, TRPML1 and TFEB pathways have been also involved in mTORC1 reactivation during prolonged starvation^74,90^, a mechanism that may limit the deleterious effects of excessive autophagy.

Overall, our data, together with previously published observations, indicate that the physiological role of TRPML1 during autophagy is more complex than expected^23^. Thus, our results suggest that TRPML1 can use multiple pathways to modulate autophagy and lysosomal biogenesis, indicating that this channel is an important hub for cell metabolism and a promising therapeutic target for a variety of disease conditions.

## Methods

### Drugs and cellular treatments

The following drugs were used to perform the assays: Torin1 (1 µM, Tocris Bioscience, 3 h); BAPTA-AM (10 µM, Thermo Fisher, cell was pre-treated for 30 min and then used in co-treatment with MK-683 for additional 30 min); EGTA-AM (10 µM, Thermo Fisher, cell was pre-treated for 30 min and then used in co-treatment with MK-683 for additional 30 min); MK6-83 (30 µM for 30 min or otherwise indicated in the text, Tocris or Dr C. Grimm from Ludwig Maximilian University); ML-SA1 (30 µM, Sigma, 30 min or otherwise indicated in the text); ML2-SA1/EVP22 (30 µM, gift from Professor Grimm C. from Ludwig Maximillian University of Munich, 30 min or otherwise indicated in the text); SN-2 (30 µM, gift from Professor Grimm C. from Ludwig Maximillian University of Munich, 30 min or otherwise indicated in the text); Bafilomycin A1 (100 nM, Sigma, 3 h); STO-609 (20 µM, Sigma, cells were pre-treated for 30 min and then used in co-treatment with MK-683 for additional 30 min); wortmannin (5 µM, Sigma, 30 min); SAR405 (0.5 µM, ApexBio, 30 min); DM (40 µM, Tocris, 30 min); MRT-68921 (30 µM, Tocris Bioscience, cells were pre-treated for 30 min and then used in co-treatment with MK-683 for additional 30 min) ACT-D (0.1 µg mL^−1^, Sigma, 3 h); Vinblastine (20 µM, Sigma-Aldrich, cells were pre-treated for 2.5 h and then used in co-treatment with MK-683 for additional 30 min). The starvation medium was prepared with HBSS (Thermo fisher), 10 mM HEPES (Sigma), for amino acid starvation medium we used RPMI without amino acid from US Biological (R9010-01) supplemented with 10% dialyzed fetal bovine serum (Thermo fisher).

### Cell culture and transfection

ARPE-19 (retinal pigment epithelium cell line), HEK-293, and HeLa M cells were purchased from American Type Culture Collection (ATCC) and were cultured in Dulbecco’s modified Eagle’s medium (DMEM) F12 and DMEM, respectively, supplemented with 10% fetal bovine serum, 200 µM l-glutamine, 100 µM sodium pyruvate, 5% CO_2_ at 37 °C. In addition to other cell lines, ARPE-19 cells were chosen to add a diploid non-transformed human cell line to confirm our data. ARPE-19 TRPML1-KO cells were generated by Dr J. Monfregola at TIGEM (Naples); ARPE-19 TRPML2 KO cell was generated by Dr A. Amabile at San Raffaele Institute (Milan); both of them were cultured in DMEM F12 supplemented with 10% fetal bovine serum, 200 µM l-glutamine, 100 µM sodium pyruvate, 5% CO_2_ at 37 °C.

HAP-1 (TRPML1-KO, TRPML3-KO, and Ctr) cells were purchased from Horizon and were cultured in IMDM supplemented with 10% fetal bovine serum, 200 µM l-glutamine, 100 µM sodium pyruvate, 5% CO_2_ at 37 °C. Control (CTR)  human patient fibroblasts were provided by Dr N. Brunetti (TIGEM); GM02526 and GM02527 were purchased from Coriell Institute and were cultured in DMEM supplemented with 15% fetal bovine serum, 200 µM l-glutamine, 5% CO_2_ at 37 °C. Hek-293 DFCP GFP was a gift of Dr Ktistakis and were cultured in DMEM supplemented with 10% fetal bovine serum, 200 µM l-glutamine, 100 µM sodium pyruvate, 0.8 mg mL^−1^ of G418, 5% CO_2_ at 37 °C. MEF WT and MEF FIP200 KO were a gift of Dr C. Settembre from TIGEM (Naples) and were cultured in DMEM supplemented with 10% fetal bovine serum, 200 µM l-glutamine, 5% CO_2_ at 37 °C. HeLa LC3 tandem were generated by stable transfection of Hela M cells with LC3 tandem vector mRFP-EGFP-LC3. HeLa TFEB/TFE3-KO was generated from Dr Youle R.J. from National Institutes of Health, Bethesda. U2OS LC3-GFP was provided by Dr Paolo Grumati (Tigem, Naples) and cultured in DMEM supplemented with 10% fetal bovine serum, 200 µM l-glutamine, 5% CO_2_ at 37 °C.

Cells were transfected for 24 h with the sequent plasmids: pDS-RED-C1-Beclin1 plasmid purchased at Addgene (Plasmid #24405); TRPML1, TRPML2, and TRPML3 purchased at Origene (respectively plasmid number RC201010, RC211195, and RC224697); TRPML1-DDKK non-conducting pore mutant was a kind gift of professor Haoxing Xu from University of Michigan, TRPML1 GFP plasmid was generated by subcloning the TRPML1 coding sequence from RC201010 in a pEGFP-C3 plasmid. HeLa cells were transfected using TransIT®-LT1 transfection reagent (Mirus) in accordance to the manufacturer’s protocol; ARPE-19 cells were transfected using Lipofectamine LTX and Plus reagent (Thermo Fisher) in accordance to the manufacturer’s protocol.

Cells were silenced with 40 nM of siRNA against *TRPML1* (sequence: 5′-CCUUCGCCGUCGUCUCAAA-3′); TRPML1 oligo #2 (sequence: 5′-AUCCGAUGGUGGUUACUGA-3′); *TRPML1* oligo #3 (sequence: 5′-GAUCACGUUUGACAACAAA-3′); *CaMKK*β (Sequence: 5′-GGCACAUCAAGAUCGCUGA-3′—oligo#1 in Supplementary Fig. 6a, 5′-UGACAAUACCUACUAUGCA-3′—oligo#2 in Supplementary Fig. 6a, 5′-CAAAGGCAUCGAGUACUUA-3′), *CaMKI* (5′-AUACAGCUCUAGAUAAGAA-3′—oligo#1 in Supplementary Fig. 6a, 5′-AGAUUUUGAAGGCCGAGUA-3′—oligo#2 in Supplementary Fig. 6a, 5′-CCAUAGGUGUCAUCGCCUA-3′), *VPS34* (5′-GAGAUGUACUUGAACGUAA-3′, 5′-GCAUGGAGAUGAUUUACGU-3′, 5′-GCUUAGACCUGUCGGAUGA-3′), *ULK1* (sequence: 5′-GCAUCGGCACCAUCGUCUA-3′—oligo#1 in Supplementary Fig. 6a, 5′-GCAUGGACUUCGAUGAGUU-3′—oligo#2 in Supplementary Fig. 6a, 5′-CGCCUGUUCUACGAGAAGA), *ULK2* (5′-GCAGAUUAUUUGCAAGCGA-3′ —oligo#1 in Supplementary Fig. 6a, 5′-GCUCGUUACCUACAUAGUA-3′, 5′-GAAUCUGAACGAACGAUAU-3′—oligo#2 in Supplementary Fig. 6a, ATG13 (L-020765-01 from Dharmacon), *Beclin1* (siGENOME *BECN1* siRNA - Pool Catalog Number M-010552-01, from Dharmacon), *TRPML2* (sequence: 5′-GGUUAUUUCCAGGCAUAUA-3′, 5′-GCUCUAAGGUUACGGAAGA-3′, 5′-GGAAUGCAGUAGCAAAGAA-3′), *TRPML3* (sequence: 5′-CAUUAGAUCUGUGAUUAGA-3′, 5′-GACUUUACUCUGACUAUAA-3′, 5′-GAGUCUAACUAGUUAUGAU-3′);

*AMPK* (sequence: 5′-GGAUCCAUCAUAUAGUUCA-3′—oligo#1 in Supplementary Fig. 6a, 5′-GAGUCUACAGUUAUACCAA-3′, 5′-CGGGAUCAGUUAGCAACUA-3′—oligo#2 in in Supplementary Fig. 6a), *Beclin1* (Santa Cruz Biotechnology, Inc., BECN1 siRNA (h):sc-29797)—pool2 in Supplementary Fig. 6m), *Syntaxin-17* (sequence: 5′-GGAGAAGAUUGACAGCAUU-3′, 5′-CCUUUGACCAGAUCCAUGA-3′, 5′-GAGUUUGACUCAGAUAUAU-3′) for 72 h using Lipofectamine RNAimax (Thermo Fisher) according to the manufacturer’s protocol. In Supplementary Fig. 4 are reported all control experiment (qPCR or western blottings) performed for checking the reduction of gene expression in KO and silenced cells.

### Generation of ARPE-19 CrispCa9

Generation of ARPE-19 CrispCa9 *MCOLN1* KO cell lines. ARPE-19 (ATCC CCRL-2320) cells carrying a homozygous deletion of a C were generated by using the CRISPr/Cas9 system. The gRNA sequence (5′-CCCTGCGACAAGTTTCGAGCCA-3′) with low off-target score have been selected using the http://crispor.tefor.net/crispor.py tool. An “ALL in One” vector expressing Cas9, the specific gRNA, and GFP was obtained from SIGMA (CAS9GFPP). The CAS9GFPP was nucleofected in ARPE-19 cells using the Amaxa Cat No VCA-1003 and transfected GFP-positive cells were fluorescence-activated cell sorted (FACS) into 96-well plates to obtain single-cell-derived colonies carrying the INDEL mutations. Upon genomic DNA extraction and DNA Sanger sequencing, a cell clone carrying the c.159delC was selected and expanded (Supplementary Fig. 9d). Generation of ARPE-19 CrispCa9 *MCOLN2* KO cell line. ARPE-19 cell line was electroporated with plasmid encoding spCas9 and sgRNA targeting exon 1 of the *TRPML2* gene (sgRNA: 5′-TGGCTCGGTATTTTTCACAA GGG-3′, in bold PAM sequence). Seven days post-electroporation, cells were analized by Surveyor Nuclease assay (Surveyor Mutation Detection kit, IDT Technology, catalog number 706020) to measure levels of NHEJ at *TRPML2* locus. 2 weeks after electroporation single-cell clones were derived from bulk population through FACS sorting. Genomic DNA of individual clones were extracted and amplified with the following primers for the *TRPML2* locus (F: 5′-CTTGTGGTAAGGGAAAAACCAA-3′; R: 5′-GGAGAGGCTTTCCTGGATATTT-3′). PCR products were cloned in TOPO™ TA Cloning™ Kit (Thermo Fisher catalog number K457501) and sequenced. Selected clone presented at sgRNA cutting site Indel/Mutationa at both alleles (T insertion) that introduce a premature stop codon (Supplementary Fig. 9e). HAP-1 KO cells line: cell lines were purchased from Horizon. HAP-1 *MCOLN1* KO: https://www.horizondiscovery.com/human-mcoln1-knockout-cell-line-hzghc002537c011. Guide RNA sequence 5′-TGCGACAAGTTTCGAGCCAA-3′. Targeted region: exon 2, mutation 5 bp deletion. HAP-1 *MCOLN3* KO: https://www.horizondiscovery.com/human-mcoln3-knockout-cell-line-122bp-deletion. Guide RNA sequence: 5′-CAGCTATACAATGTCTCCGT-3′. Targeted region: exon 4, Mmutation 122 bp deletion.

### Antibodies and western blotting

For western blottings, the following antibodies were used: B-Actin (Santa Cruz SC 47778, 1:4000), WIPI2 (Abcam, catalog number ab105459, 1:1000), LC3 (Novus NB100-2220, 1:1000), ULK1 (Cell Signalling, catalog number 8054 1:1000), Phospho-ULK1 (Ser757) (Cell Signalling, catalog number 6888 1:1000), Phospho-ULK1 (Ser555) (Cell Signalling, catalog number 5869 1:1000), p70 S6 Kinase (Cell Signalling, catalog number 2708 1:1000), Phospho-p70 S6 kinase (Thr389) (Cell Signalling, catalog number 9205 1:1000), Phospho-AMPKα (Thr172) (Cell Signalling, catalog number 2535 1:1000), AMPKα (Cell Signalling, catalog number 5831 1:1000), Phospho-ACC (Ser79) (Cell Signalling, catalog number 3661 1:1000), ACC (Cell Signalling, catalog number 3676 1:1000), CaMK1 (Abcam ab68234 1: 1000), CaMKK (Abcam Ab174289 1:1000), Phospho-BECLIN (Ser15) (Cell Signalling, catalog number 13825 1:1000), VINCULIN (Sigma, catalog number 9264), VPS34 (Z-R015, Echelon), VPS34 (Santa Cruz Biotechnology, Inc., Ab365404, 1:1000), ATG14 (Cell Signalling, catalog number 5504,1:1000), BECLIN1 (Cell Signalling, catalog number 3495, 1:1000), and Monoclonal ANTI-FLAG® M2 antibody (Sigma F3165 1:4000). Total cell lysate was prepared by solubilization in TRIS HCl 10 mM pH 8.0 and 0.2% SDS supplemented with protein and phosphatases inhibitor (Sigma). Protein concentration was determined by the Bradford method. After SDS-polyacrylamide gel electrophoresis (PAGE) and immunoblotting, the protein recognized by the specific antibody were visualized by chemiluminescence methods (Luminata Crescendo Western HRP substrate, Millipore) using peroxidase-conjugated anti-rabbit or anti-mouse secondary antibodies (Millipore). Membranes were developed using a Chemidoc UVP imaging system (Ultra-Violet Products, Ltd) and densitometric quantification was performed in unsaturated images using ImageJ (NIH). All uncropped and unprocessed scans of western blottings are provided as Supplementary Fig. 11.

### VPS34 immunoprecipitation and VPS34 kinase assay

HeLa cells were treated with DMSO (dimethylsulfoxide), MK6-83, or ML-SA1 for 30 min. VPS34 immunoprecipitation was performed in accordance with the Echelon protocol (Z-R015 from Echelon). Two milligrams of total protein lysate was incubated overnight with 2 μg mg^−1^ of protein of VPS34 antibody Z-R015. Day after immunoprecipitated complexes were incubated 2 h with protein A/G PLUS-Agarose (sc-2003 Santa Cruz). The kinase assay was performed on VPS34 immunoprecipitated enzyme using the Class III PI3K Elisa Kit (K-3000 Echelon) following the instruction for beads conjugated enzyme. PI3P production was performed at 30 °C for 40 min in constant shaking of 1100 r.p.m. Produced PI3P was collected by centrifugation from the beads and used for colorimetric detection on Elisa plate. Beads were resuspended in 35 µl of 2× Laemmli Sample Buffer and boiled at 95 °C for 5 min, and run on SDS-PAGE to visualize the VPS34 complex by immunoblot.

### Immunofluorescence

For immunofluorescence, the following antibodies were used: WIPI2 (Abcam, catalog number ab105459, 1:1000), Atg16L1 (D6D5, Cell Signaling, catalog number 8089, 1:200), LC3 (MBL PM036 1:400), LAMP1 (Santa Cruz, catalog number sc-20011, 1:400), TFEB (Cell Signalling, catalog number 4240 1:200), Phospho-S6 Ribosomal Protein (Ser235/236) (Cell Signalling, catalog number 9865 1:400), and FLAG M2 (Sigma, catalog number F3165). For WIPI2 and Atg16L1 staining, cells were fixed with ice-cold methanol for 5 min on ice and permeabilized in 0.1% (w/v) saponin, 0.5% (w/v) bovine serum albumin (BSA), and 50 mM NH4Cl in phosphate-buffered saline (PBS) (blocking buffer saponin). For LC3 and TFEB, cells were fixed in paraformaldehyde 4% for 20 min. For LC3, cells were permeabilized with blocking buffer saponin, whereas for TFEB cells permeabilized in 0.1% (w/v) Triton-X, 1% (w/v) horse serum, and 1% (w/v) BSA in PBS. Cells were incubated with the indicated primary antibodies for 1 h and subsequently incubated with secondary antibodies for 45 min.

The PX-594-conjugated antibody (1:200) was kindly provided by Dr. Ian Ganley from the University of Dundee. The staining was performed in accordance with the protocol described by Munson and Ganley^44^.

For confocal imaging, the samples were examined under a Zeiss LSM 800 confocal microscope. Optical sections were obtained under a ×63 or ×40 immersion objective at a definition of 1024 × 1024 pixels (average of 8 or 16 scans), adjusting the pinhole diameter to 1 Airy unit for each emission channel to have all the intensity values between 1 and 254 (linear range). For high-content images, we use OPERA system from PerkinElmer.

For image analysis, we used Columbus 2.6.0.127073 (built at 03:56 on 05/02/19) released by PerkinElmer. This online platform is based on Harmony High-Content Imaging and Analysis Software, which provides an easy quantification of complex cellular phenotypes. All the images were processed as follows: before starting the analysis sequence, the images were first segmented into the nuclei and cytoplasm using the Find Nuclei building block on the Hoechst channel and the Find Cytoplasm on the Cell Mask channel. To detect the spots (WIPI, LC3, and LAMP), the Find spots building block was applied to the spot channel. Then the Select population building block was used to select spots of certain intensity. The intensity and the morphology of each spot were calculated by adding the building blocks Calculate Intensity Properties and Calculate Morphology Properties. Finally, with define results building block we calculate the number of the spot for each cell.

### Immuno-electron microscopy

Cells were fixed in a mixture of 4% paraformaldehyde and 0.05% glutaraldehyde prepared in 0.2 M HEPES. Then, the cells were washed and incubated with the blocking/ permeabilizing mixture (0.5% BSA, 0.1% saponin, 50 mM NH_4_Cl) for 30 min and subsequently with the primary antibody against WIPI2, diluted 1:100 in blocking/permeabilizing solution, overnight. The following day the cells were washed and incubated with the secondary antibody, the anti-rabbit Fab’ fragment coupled to 1.4 nm gold particles, diluted 1:50 in blocking/permeabilizing solution, for 2 h. After this incubation with the antibodies, the gold-enhancement reaction has to be performed to increase the size of the 1.4 nm gold particles. Then samples were post-fixed in a mixture of osmium tetroxide and potassium ferrocyanide, dehydrated in ethanol and acetone, and embedded in epoxy resin as described previously (Polishchuk et al.^61^). Thin, 60 nm serial sections were cut using a Leica EM UC7 ultramicrotome. Electron microscopic images were acquired using a FEI Tecnai-12 electron microscope (FEI, Eindhoven, Netherlands) equipped with a VELETTA CCD digital camera (Soft Imaging Systems GmbH, Munster, Germany).

### RNA extraction and qPCR

Total RNA was extracted from cells using RNeasy Plus Mini Kit (Qiagen). Reverse transcription was performed using QuantiTect Rev Transcription Kit (Qiagen). Real-time quantitative reverse-transcription PCR (qRT-PCR) was performed using the LightCycler® System 2.0 (Roche Applied Science). *HPRT* was used for qRT-PCR as reference gene. The parameters of real-time qRT-PCR amplification were according to Roche recommendations. The following primers were used in this study: *HPRT* fw: 5′-TGGCGTCGTGATTAGTGATG-3′, rev: 5′-AACACCCTTTCCAAATCCTCA-3′; *TRPML1* fw: 5′-GAGTGGGTGCGACAAGTTTC-3′, rev: 5′-TGTTCTCTTCCCGGAATGTC-3′; *WIPI2* fw: 5′-TACCTGCCTTCCCAAGTGAC-3′, rev: 5′-AGCGAGCAGATGTTTTTGTG-3′, *ATP6V0E1*; fw: 5′-cattgtgatgagcgtgttctgg-3′, rev: 5′-AACTCCCCGGTTAGGACCCTTA-3′, *ATP6V1H*; fw: 5′-GGAAGTGTCAGATGATCCCCA-3′, rev: 5′-CCGTTTGCCTCGTGGATAAT-3′, *TFEB*; fw: 5′-CAAGGCCAATGACCTGGAC-3′, rev: 5′-AGCTCCCTGGACTTTTGCAG-3′, *DPP7*; fw: 5′-GATTCGGAGGAACCTGAGTG-3′, rev: 5′-CGGAAGCAGGATCTTCTGG-3′, *TPP1*; fw: 5′-GATCCCAGCTCTCCTCAATAC-3′, rev: 5′-GCCATTTTTGCACCGTGTG-3′, *NEU1*; fw: 5′-TGAAGTGTTTGCCCCTGGAC-3′, rev: 5′-AGGCACCATGATCATCGCTG-3′; *ATG135*′-GACCTTCTATCGGGAGTTTCAG-3′, rev: 5′-GGGTTTCCACAAAGGCATCAAC-3′; *ULK1* fw: 5′-AGGTGGCCGAGCTACTGTC-3′, rev: 5′-GGCCTTGTACACCTCATTC-3′; *ULK2* fw: 5′-GGTTGGTACCSTTCCTGAGC-3′; rev: 5′-AGAGACTGTGGGGACTGAG-3′; *Beclin1* fw: 5′-AGCCCCTGAAACTGGACAC-3′, rev: 5′-TCTTCCTCCTGGGTCTCTCC-3′; *CaMK1* fw: 5′-TGTCATCGCCTACATCTTGC-3′, rev: 5′-CAAACTCGTACTCGGCCTTC-3′; *CaMKK*β fw: 5′-TCAAACCTTCCAACCTCCTG, rev: 5′-TTGCTCACACCAAAGTCAGC-3′; *TRPML3* fw: 5′-GAAGTTCTGGGCTCGAGGTAG-3′, rev: 5′-GATTCCCAACGGAGACATTG-3′; *TRPML2* fw: 5′-ATGGCACATCGTGATTCTGA-3′, rev: 5′-CGTCTGGCTCGGTATTTTTC-3′; *AMPKα* fw: 5′-GTATGCAGGCCCAGAGGTAG-3′, rev: 5′-GAGTTGGCACATGGTCATCA-3′; *MAP1* fw: 5′-CGGAGAAGACCTTCAAGCAC-3′, rev: 5′-CTGCTTCTCACCCTTGTATCG-3′.

### Statistics and reproducibility

Sample sizes and reproducibility for each figure are denoted in the figure legends. Immunofluorescence experiments were repeated independently 3 times and at least 50 cells were analyzed from a single experiment. Representative images are shown in Figs. 1a, b, d, e, 2a–e, 3a–e, 4a–d, f, g, 5a, and 6a–e, and in Supplementary Figs. 1, 2a, b, e, 3, 4b, c, e, f, h, 5a, c, d, 6a, m, 7a–e, 8a–c, 9, and 10c. Western blotting and immunoprecipitation experiments were repeated at least three times and representative blots are shown in Figs. 1c, 4e, h, i, and 5b, c, and in Supplementary Figs. 2c, d,  3a, 4e–g, 5b, e, 6l, 7f, g, and 10a. Most data are presented as the means ± SD unless otherwise specified. Statistical comparisons were made using Student’s *t*-test when comparing two groups.

## Supplementary information

Supplementary Information

## Data Availability

The datasets generated and/or analyzed during the current study are available from the corresponding author on reasonable request. The source data underlying Figs. 1a, b, d, e, 2, 3, 4a–d, f, g, 5a, and 6a–f are provided as a Source Data file.

## Source data

Source Data
