# Supplementary material for: TRPML1 links lysosomal calcium to autophagosome biogenesis through the activation of the CaMKKβ/VPS34 pathway
Source: Nat Commun. 2019 Dec 10;10:5630. doi: 10.1038/s41467-019-13572-w (PMC6904751; doi:10.1038/s41467-019-13572-w)

Cover Page – Supplementary Information

TRPML1 links lysosomal calcium to autophagosome biogenesis through the activation of the CaMKKβ/VPS34 pathway

Scotto Rosato A., et al.

Supplementary Figure 1

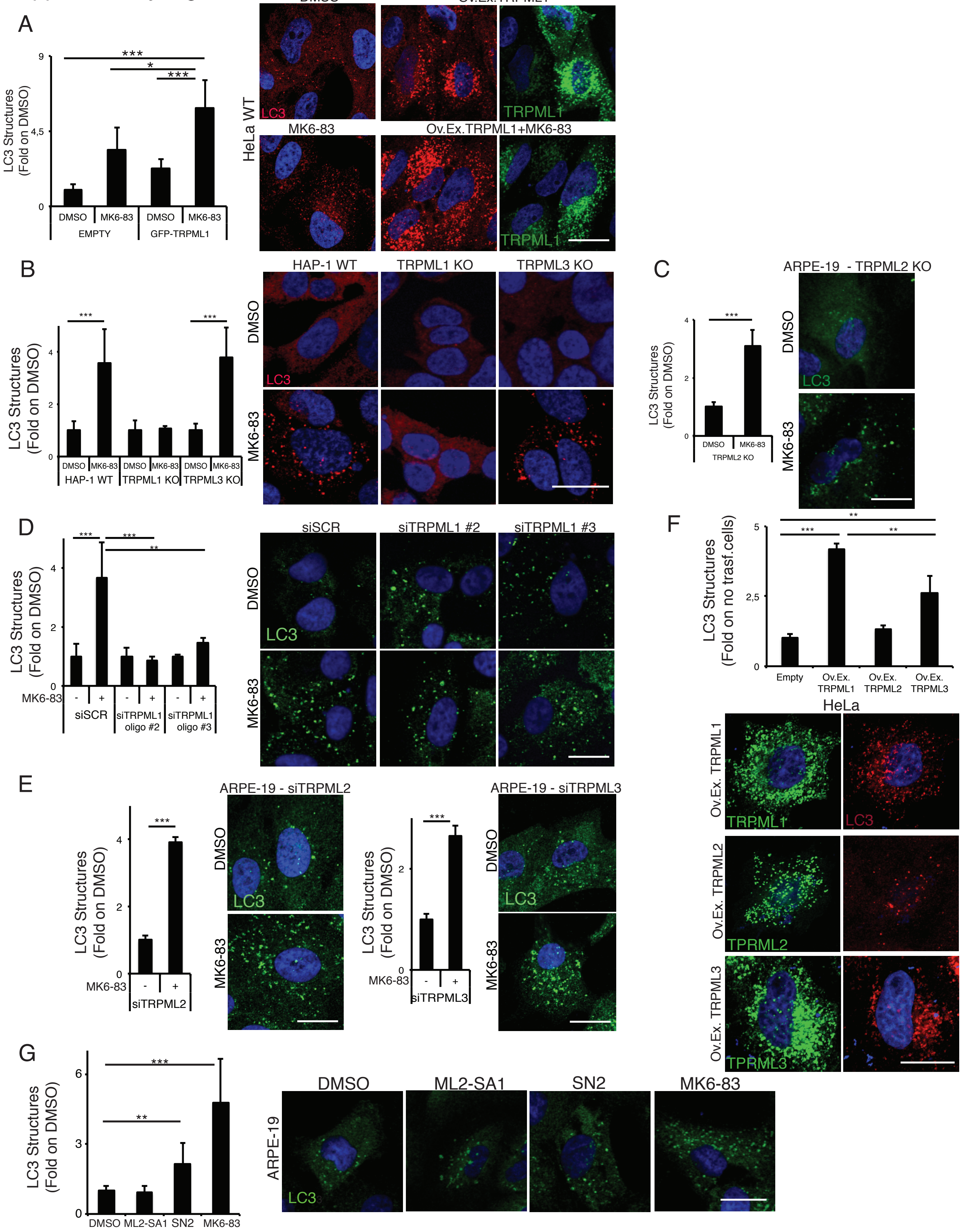

Supplementary figure 1.

(A) Representative images of LC3 in HeLa cells transfected with TRPML1-GFP, upon MK6-83 treatment . Plots show LC3 puncta quantification as fold induction to cells treated with DMSO. Values are means ± s.d. of n>1000 cells pooled from 3 independent experiments. (B) Representative images of LC3 in HAP-1 WT, TRPML1 KO and TRPML3 KO cells upon MK6-83 treatment . Plots show LC3 puncta quantification as fold induction to DMSO treated cells. Values are means ± s.d. of n>1000 cells pooled from 3 independent experiments. (C) Representative images of LC3 in ARPE-19 - TRPML2 KO upon MK6-83 treatment . Plots show LC3 puncta quantification as fold induction to DMSO treated cells. Values are means ± s.d. of n=300 cells pooled from 3 independent experiments. (D) Representative images of LC3 in ARPE-19 silenced with two different oligos targeting TRPML1 (#2 and #3), upon treatment with MK6-83. Plots show LC3 puncta quantification as fold induction to DMSO treated cells. Values are means ± s.d. of n>1000 cells pooled from 2 independent experiments. (E) Representative images of LC3 in ARPE-19 cells silenced with a pool of three siRNA molecules targeting respectively TRPML2 and TRPML3 (siTRPML2, siTRPML3), upon treatment with MK6-83. Plots show LC3 puncta quantification as fold induction to DMSO treated cells. Values are means ± s.d. of n>1000 cells pooled from 3 independent experiments and n>1000 cells pooled from 3 independent experiments. (F) Representative images of LC3 (red) in ARPE-19 cells transfected with a vector encoding flag-tagged (green) TRPML1 (upper panel), TRPML2 (middle panel) and TRPML3 (lower panel). Plots show LC3 puncta quantification as fold induction to cells transfected with empty vector. Values are means ± s.d. of n=165 cells pooled from 2 independent experiments. (G) Representative images of LC3 in ARPE-19 cells treated with TRPML2 agonist ML2-SA1, TRPML3 agonist SN2 and MK6-83. Plots show LC3 puncta quantification as fold induction to cells treated with DMSO. Values are means ± s.d. of n>1000 cells pooled from 3 independent experiments. Scale bar: 20 μm. P-values calculated by two tails Student's t-test. * represents p-value < 0,05; ** represents p-value < 0,01; *** represents p-value<0.001.

Supplementary Figure 2

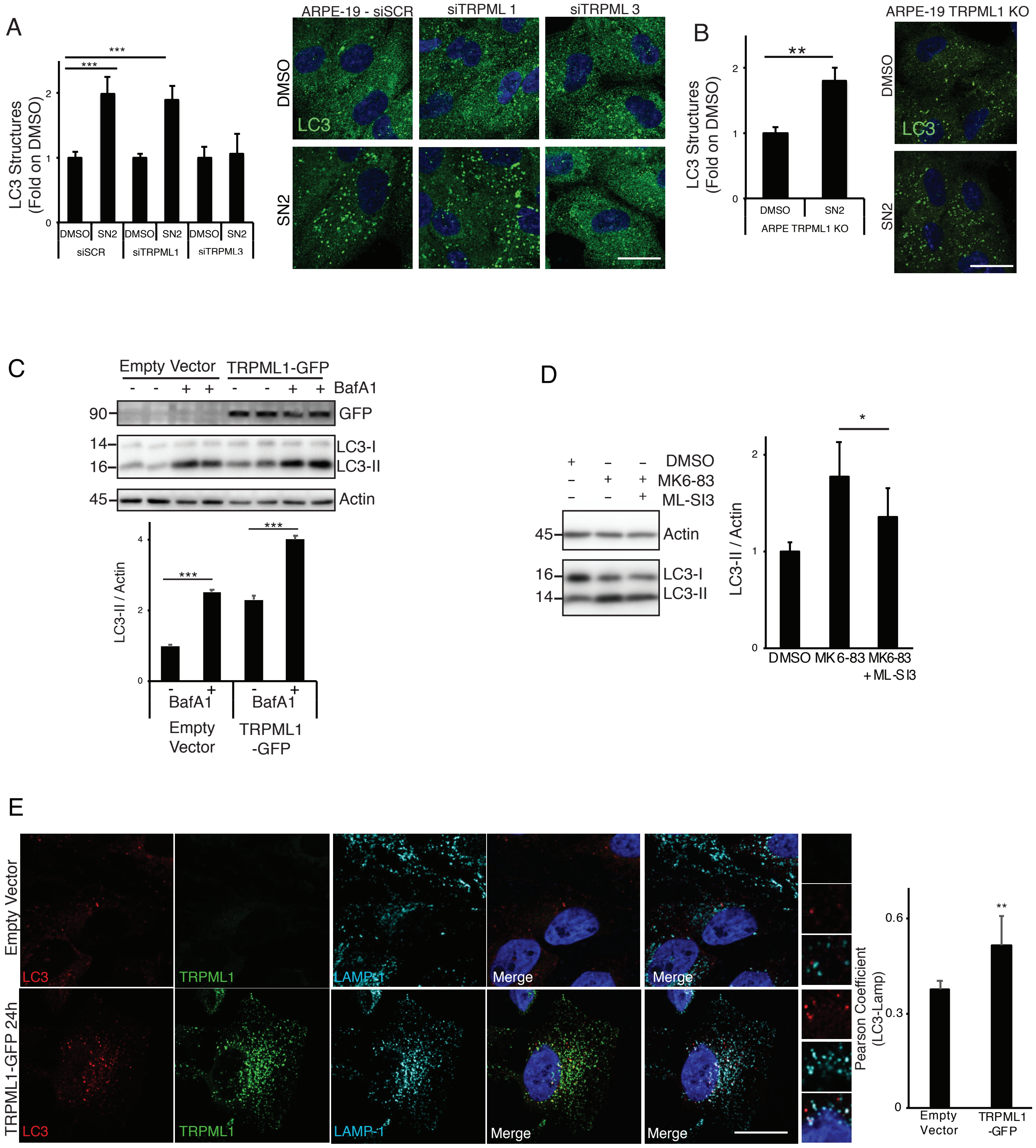

Supplementary figure 2.
(A) Representative confocal images of endogenous LC3 in ARPE-19 silenced for TRPML1 and TRPML3, treated with SN2. Plots show LC3 puncta quantification as fold induction to cells treated with DMSO. Values are means ± s.d. of n=750 cells pooled from 3 independent experiments. (B) Representative confocal images of endogenous LC3 in ARPE-19 TRPML1 KO, treated with TRPML3 agonist SN2. Plots show LC3 puncta quantification as fold induction to cells treated with DMSO. Values are means ± s.d. of n=390 cells pooled from 3 independent experiments. (C) Representative image of immunoblot analysis of endogenous LC3 in HeLa cells transfected with Empty vector or TRPML1 for 16 h in DMSO alone or in co-treatment with bafilomycin A1 (BafA1). The plot below shows the densitometry of LC3II band normalized to Actin as fold on Empty vector in DMSO. Data in the graphs are mean values ± s.d. of n=6 lysates per condition pooled from 3 independent experiments. (D) Representative image of immunoblot analysis of endogenous LC3 in ARPE-19 cells treated with MK6-83 alone or in co-treatment with ML-SI3. The plot below shows the densitometry of LC3II band normalized to Actin as fold on DMSO. Data in the graphs are mean values ± s.d. from 3 independent experiments. (E) Representative confocal images of endogenous LC3 (red), LAMP-1 (Cyan) in ARPE-19 overexpressing TRPML1-GFP (green) for 24 hours. The plot shows Pearson correlation coefficient for colocalization between LC3 and LAMP-1. P-values calculated by two tails Student's t-test. Scale bar: 20 μm. * represents p-value < 0,05; ** represents p-value < 0,01; *** represents p-value<0.001.

Supplementary Figure 3

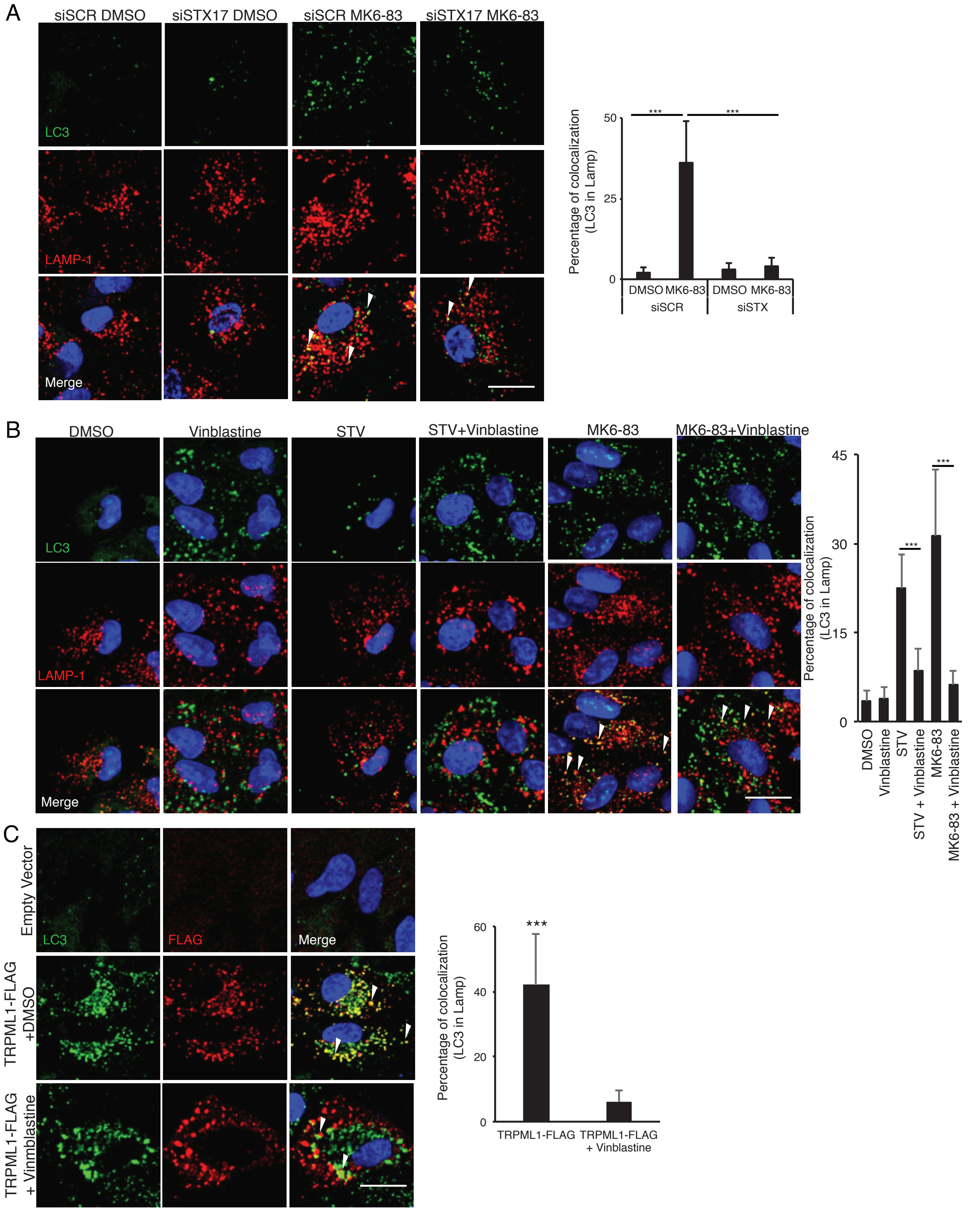

Supplementary figure 3
(A) Representative confocal images of endogenous LC3 (green) and LAMP-1 (red) in ARPE-19 WT cells silenced with a pool of three siRNA molecules targeting STX17 (siSTX17) gene and treated with MK6-83. White arrowheads indicate LC3-LAMP-1 (yellow) colocalizing spots. (B) Representative confocal images of endogenous LC3 (green) and LAMP-1 (red) in ARPE-19 treated for 30 minutes with DMSO, starvation, MK6-83 alone or in co-treatment with Vinblastine. (C) Representative confocal images of endogenous LC3 (green) in ARPE-19 transiently transfected with a vector encoding TRPML1 tagged with FLAG (TRPML1-FLAG, red) or an empty-FLAG vector. Cells were pretreated for 180 min with DMSO or Vinblastine. White arrowheads indicate LC3-LAMP-1 (yellow) colocalizing spots. Scale bar: 20μm. *** represents p-value<0.001.

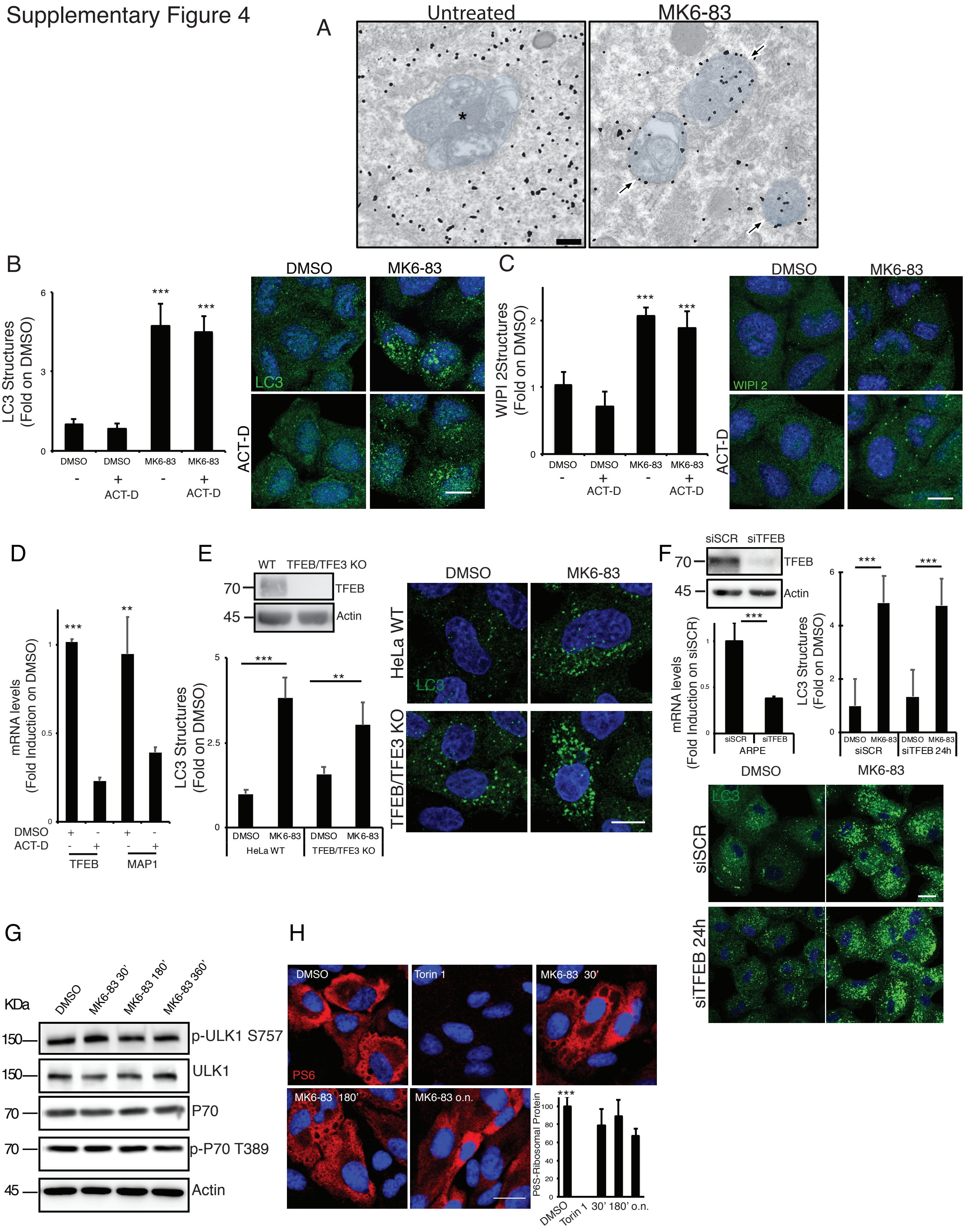

Supplementary figure 4.

(A) Representative immuno-electron microscopy images of U2Os cells stably expressing LC3-GFP labeled with an antibody against GFP. LC3 positive membrane-bound structures are indicated by arrows. Lysosome/autolysosome-like structure where LC3 signal was not detected is indicated by asterisk. Scale bar: 250 nm. (B-C) Representative confocal images of endogenous LC3 and WIPI2 in ARPE-19 WT cells treated with MK6-83 +/- Actinomycin D (ACT-D). Plots show LC3 and WIPI2 puncta quantification as fold induction to cells treated with DMSO. Values are means ± s.d. of n>1000 cells pooled from 3 independent experiments. (D) mRNA levels of TFEB and MAP1 genes upon treatment with actinomycin D (ACT-D). (E) Representative confocal images of endogenous LC3 in HeLa TFEB/TFE3 KO treated with MK6-83. Plots show LC3 puncta quantification as fold induction to cells treated with DMSO. Values are means ± s.d. of n=491 cells pooled from 2 independent experiments. Western blot shows complete depletion of TFEB in the cell lines. (F) Representative confocal images of endogenous LC3 in ARPE-19 silenced for TFEB for 24 hours and treated with MK6-83. Left plot show TFEB mRNA levels upon silencing, and western blot shows partial depletion of TFEB in the cell line. Right plot shows LC3 puncta quantification as fold induction to cells treated with DMSO. Values are means ± s.d. of n>1000 cells pooled from 3 independent experiments. (G) Representative image of immunoblot analysis of endogenous phosphorylation of mTORC1 substrates ULK1 on S757 and p70 S6 Kinase (P70) on T839, upon MK6-83 treatment (30, 180, 360 minutes) in HeLa cells. (H) Representative confocal images of endogenous phospho S6 ribosomal protein on Ser 235/236 upon treatment with Torin1, MK6-83 (30 minutes, 180 minutes and 16 hours). Plot shows the percentage of red positive cells 100% DMSO, 0% TORIN1 (n>300 cells per conditions). Scale bar A: 250 nm; B-H: 20 μm. P-values calculated by two tails Student's t-test. * represents p-value < 0,05; ** represents p-value < 0,01; *** represents p-value<0.001.

Supplementary Figure 5

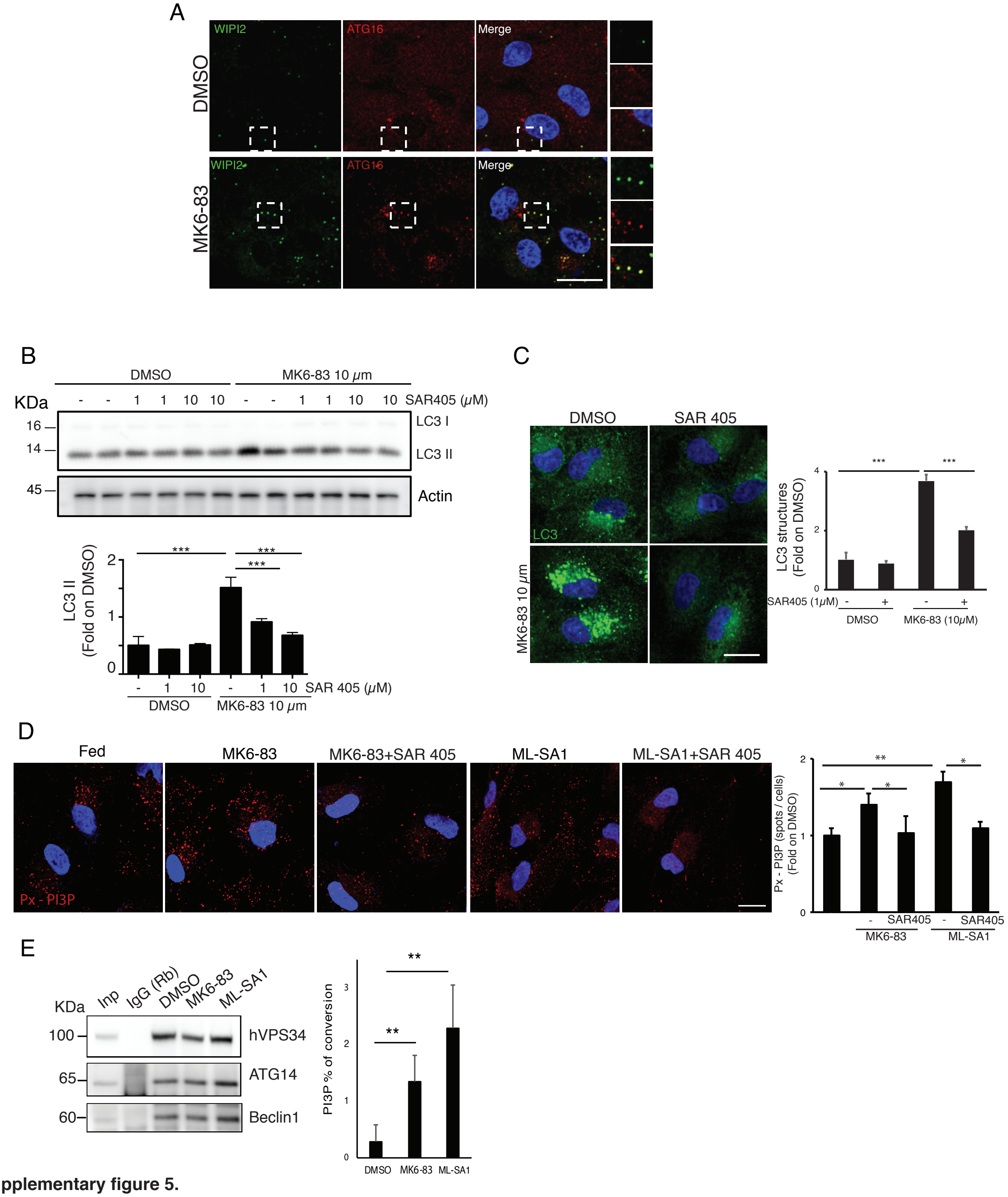

Supplementary figure 5.
(A) Representative confocal images of endogenous WIPI2 (green) and ATG16L1 (red) in ARPE-19 treated for 30 minutes with DMSO and MK6-83. (B) Representative image of immunoblot analysis of endogenous LC3 in HeLa cells treated with DMSO or MK6-83 alone or in co-treatment with SAR405. The plot below shows the densitometry of LC3II band normalized to Actin as fold on DMSO. Data in the graphs are mean values ± s.d. of n=4 lysates per condition pooled from 2 independent experiments. (C) Representative confocal images of endogenous LC3 in ARPE cells treated with DMSO or MK6-83 alone or in co-treatment with SAR405. Plot shows the quantification of LC3 positive puncta as fold induction to DMSO treated cells. Values are means ± s.d. of n>1000 cells pooled from 3 independent experiments. (D) Representative confocal images of PI3P puncta visualized by the PX domain of the p40phox subunit of the NADPH oxidase in ARPE-19 cells treated with MK6-83 or ML-SA1 alone or in combination with SAR405. Plot shows the quantification of PX positive puncta as fold induction to DMSO-treated cells. Values are means ± s.d. of n=316 cells pooled from 3 independent experiments. (E) Representative image of immunoblot analysis of immunoprecipitated endogenous VPS34 complex (VPS34, Beclin1, ATG14) in HeLa cells treated with DMSO, MK6-83 or ML-SA1. Immunoprecipitated complex was used for VPS34 kinase assay activity. Plot shows the percentage of PI3P conversion upon MK6-83 or ML-SA1 treatment. The data in the graphs on the right are mean values ± s.d., n=3 lysates per condition pooled from 3 independent experiments. P-values calculated by two tails Student's t-test. Scale bar: 20μm * represents p-value < 0,05; ** represents p-value < 0,01; *** represents p-value<0.001.

Supplementary Figure 6

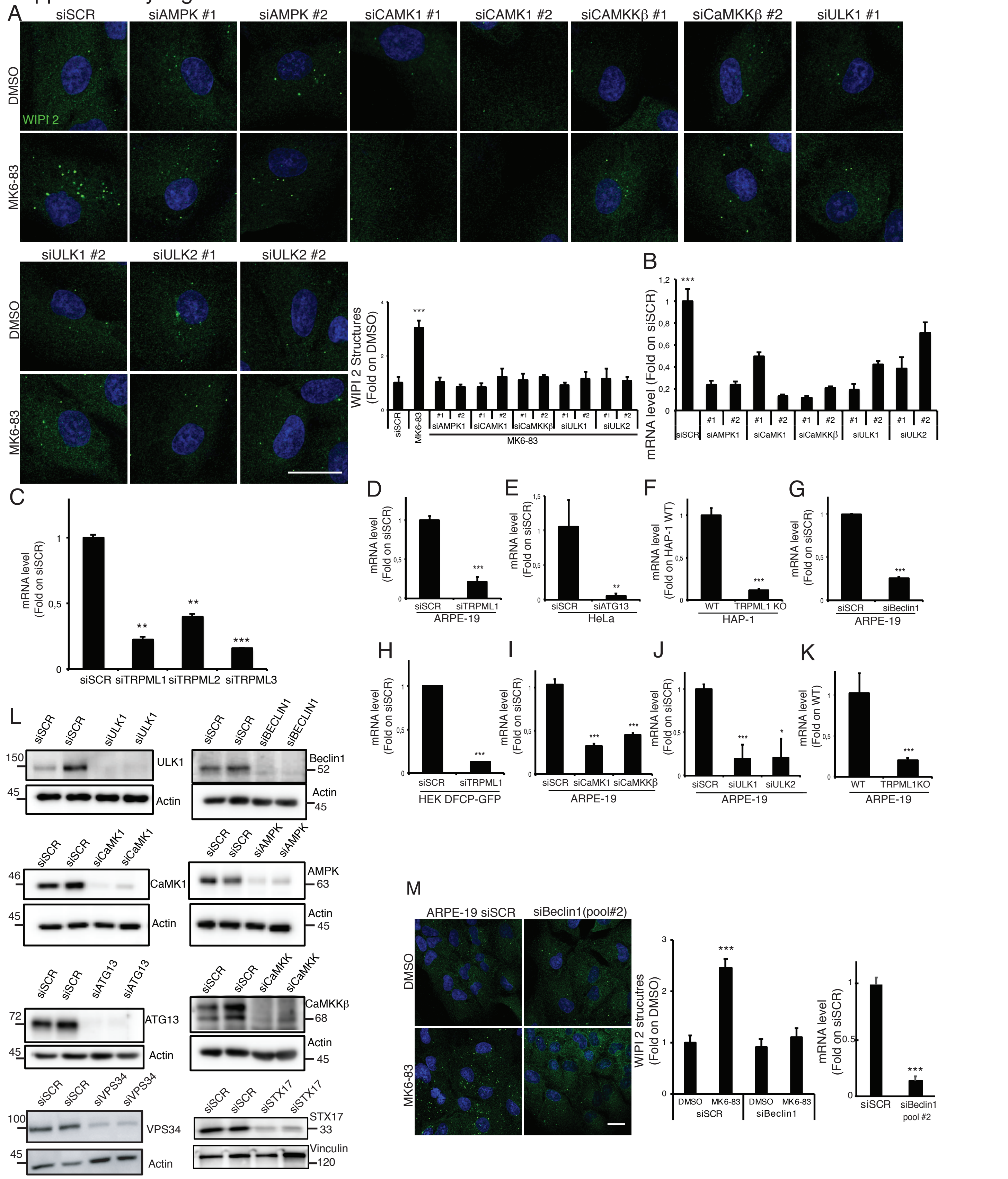

Supplementary figure 6.

(A) Representative confocal images of endogenous WIPI2 in ARPE-19 WT cells silenced with two different oligos (#1 and #2) for each gene (AMPK, CAMK1, CAMKKβ, ULK1, ULK2), and upon treatment with MK6-83. Plots show WIPI2 puncta quantification as fold induction to cells treated with DMSO. Values are means ± s.d. of n>100 cells pooled from 3 independent experiments. (B) Plots show mRNA levels of genes silenced in (A). (C) mRNA levels of TRPML1, TRPML2 and TRPML3 in ARPE-19 silenced for the corresponding gene. (D) mRNA levels of TRPML1 in ARPE-19 TRPML1-silenced cells. (E) mRNA levels of ATG13 in HeLa ATG13-silenced cells. (F) mRNA levels of TRPML1 in HAP-1 TRPML1 KO cells. (G) mRNA levels of Beclin1 in ARPE-19 Beclin1-silenced cells. (H) mRNA levels of TRPML1 in HEK DFC-GFP TRPML1-silenced cells. (I) mRNA levels of CAMK1 and CAMKKβ in ARPE-19 silenced for CAMK1 and CAMKKβ, respectively. (J) mRNA levels of ULK1 and ULK2 in ARPE-19 silenced for ULK1 and ULK2, respectively. (K) mRNA levels of TRPML1 in ARPE-19 TRPML1 KO cells. (L) Western blot show, in duplicate, the knockdown efficiency of the following proteins ULK1, Beclin1 (siGENOME, Dharmacon), CAMK1, AMPK, ATG13, CAMKK β, VPS34, STX17 silenced with a pool of three siRNA. (M) Representative confocal images of endogenous WIPI2 in ARPE-19 cells treated with siRNA molecules targeting Beclin1 followed by treatment with MK6-83. Plot shows the quantification of WIPI2 positive puncta as fold induction to DMSO-treated cells. Values are means ± s.d. of n=666 cells pooled from 3 independent experiments. Quantitative PCR showing the mRNA levels of Beclin1 in cells silenced with scramble siRNA compared to cells silenced with siRNA targeting Beclin1 (pool #2, Santa Cruz Biotechnology, Inc.). The data in the graphs are mean values ± s.d., n=3 samples per condition. P-values calculated by two tails Student's t-test. Scale bar: 20μm * represents p-value < 0,05; ** represents p-value < 0,01; *** represents p-value<0.001.

Supplementary Figure 7

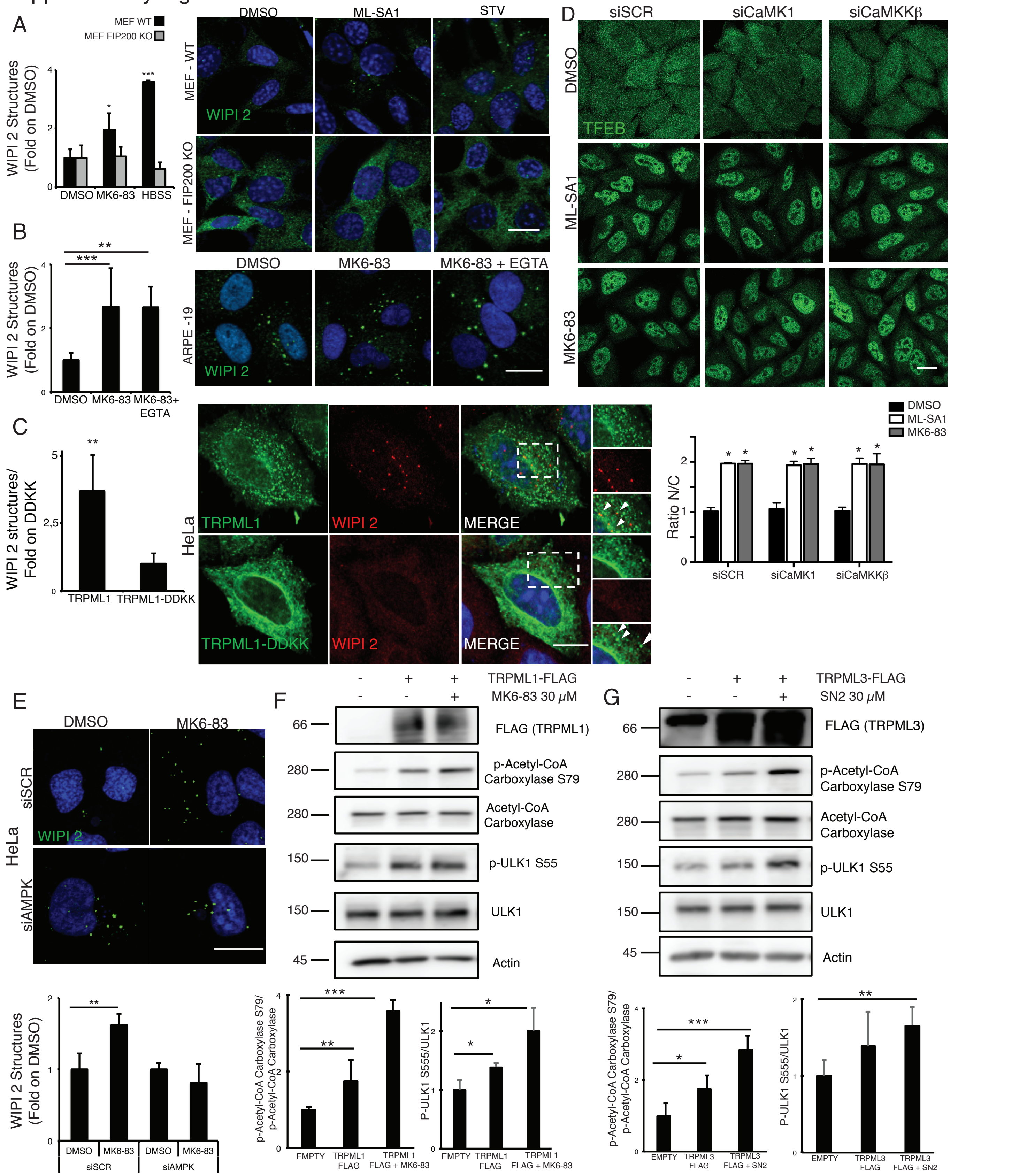

Supplementary figure 7.

(A) Representative images of WIPI2 in MEF and FIP200 KO cells, treated with ML-SA1 and starvation. Values are means ± s.d. of n=550 cells pooled from 2 independent experiments. (B) Representative images of WIPI2 in ARPE-19 WT treated with MK6-83 +/- EGTA. (C) Representative images of WIPI2 in HeLa transfected with TRPML1 or the mutant TRPML1-DDKK. Plots show the number of WIPI2 puncta per cell. Values are means ± s.d. of n=906 cells pooled from 3 independent experiments. (D) Representative images of TFEB in HeLa cells treated with a pool of three siRNA molecules targeting respectively CaMKI and CaMKKβ (siCaMKI, siCaMKKβ) followed by treatment with ML-SA1 or MK-683. The plot represents the quantification of the TFEB nuclear to cytosol ratio. (E) Representative images of WIPI2 in HeLa cells silenced with a pool of three siRNA molecules targeting AMPK (siAMPK), upon MK6-83 treatment. (F) Representative image of immunoblot analysis of phosphorylation of AMPK substrates ULK1 on S555, Acetyl-CoA Carboxylase (ACC) on S79, upon MK6-83 treatment in HeLa cells transfected with TRPML1 plasmid or an empty vector. Plot shows the densitometry of p-ULK1 S555 and p-ACC S79 on ULK1 and ACC respectively normalized on actin as fold induction to empty vector transfected cells. The data in the graphs below are mean values ± s.d., n=3 lysates per condition pooled from 3 independent experiments. (G) Representative image of immunoblot of phosphorylation of AMPK substrates ULK1 on S555, Acetyl-CoA Carboxylase (ACC) on S79, upon SN2 treatment in HeLa transfected with TRPML3 plasmid or an empty vector. Plot shows the densitometry of p-ULK1 S555 and p-ACC S79 on ULK1 and ACC normalized on actin as fold induction to empty vector transfected cells. The data in the graphs below are mean values ± s.d., n=3 lysates per condition pooled from 3 independent experiments. P-values calculated by two tails Student's t-test. (A,B,E) Plots show WIPI2 puncta quantification as fold induction to cells treated with DMSO. (B,D,E) Values are means ± s.d. of n>1000 cells pooled from 3 independent experiments. Scale bar A,B,D,E: 20 μm; C: 10 μm. *represents p-value<0,05; ** represents p-value < 0,01; ***represents p-value<0.001.

Supplementary Figure 8

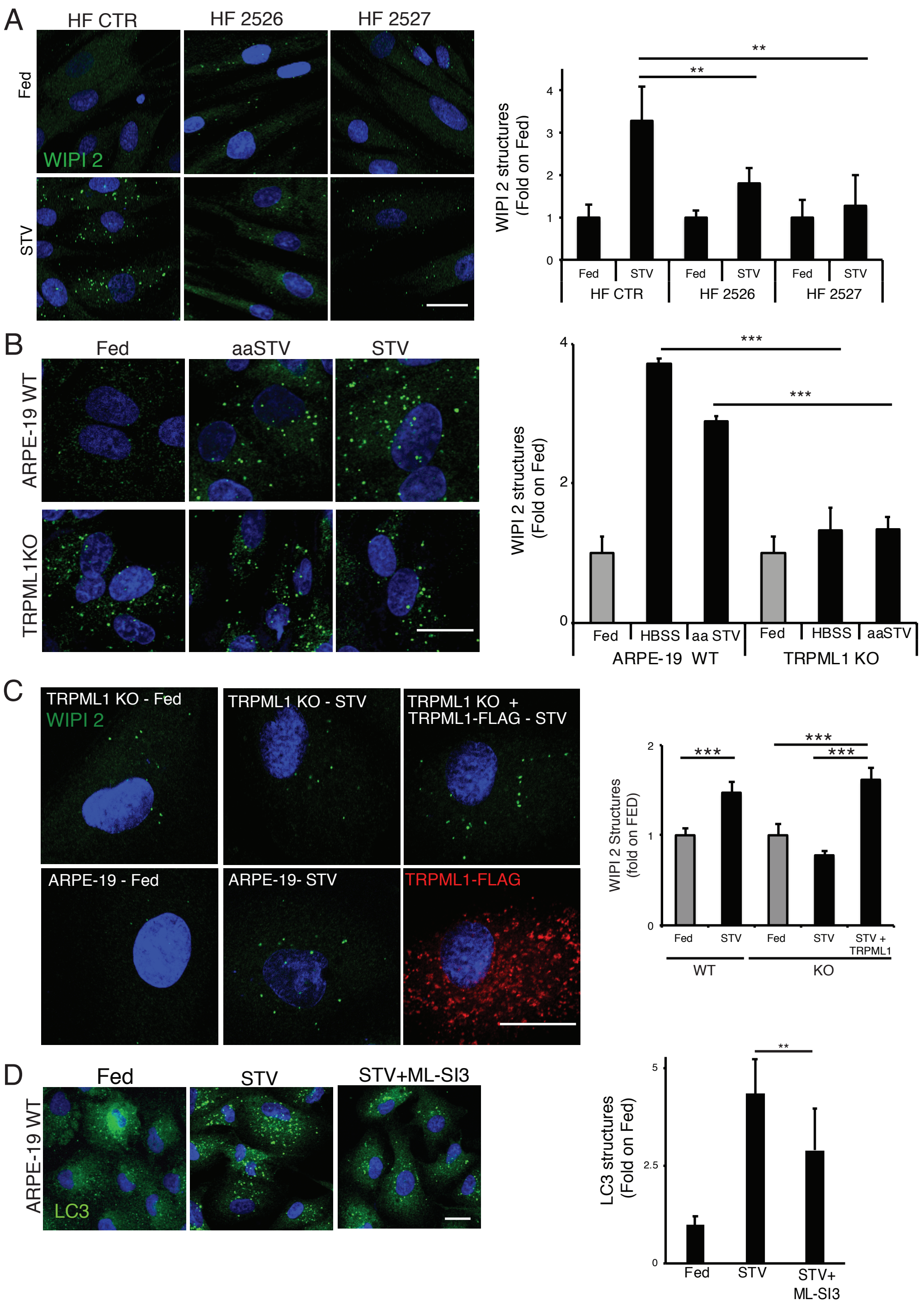

Supplementary figure 8.
(A) Representative confocal images of endogenous WIPI2 in human fibroblasts derived from a healthy individual (HF CTR) or from mucolipidosis type IV patient cell lines (GM02527, GM02526) incubated in complete medium or medium lacking of amino acids (STV). Plot shows the number of WIPI2 puncta per cell. Values are means ± s.d. of n=428 cells pooled from 3 independent experiments. (B) Representative confocal images of endogenous WIPI2 in ARPE-19 cells and TRPML1 KO cells, incubated in complete medium, medium lacking of amino acids (aaSTV) and with HBSS (STV). Plot shows the number of WIPI2 puncta per cell. Values are means ± s.d. of n=719 cells pooled from 3 independent experiments. (C) Representative confocal images of endogenous WIPI2 in ARPE-19 WT and ARPE-19 TRPML1 KO cells upon treatment with STV (amino acid). ARPE-19 TRPML1 KO cells were transiently transfected with TRPML1 wild type expression vector (in red). Plots show WIPI2 puncta quantification as fold induction to cells in fed condition. Values are means ± s.e. of n>1000 cells pooled from 3 independent experiments. (D) Representative confocal images of endogenous LC3 in ARPE-19 cells, followed by AA starvation alone or with the synthetic inhibitor of TRPML1 (ML-SI3). Plot shows the quantification of LC3 puncta as fold induction to DMSO-treated cells. Values are means ± s.d. of n>500 cells pooled from 3 independent experiments. P-values calculated by two tails Student's t-test. Scale bar: 20μm * represents p-value < 0,05; ** represents p-value < 0,01; *** represents p-value<0.001.

Supplementary Figure 9

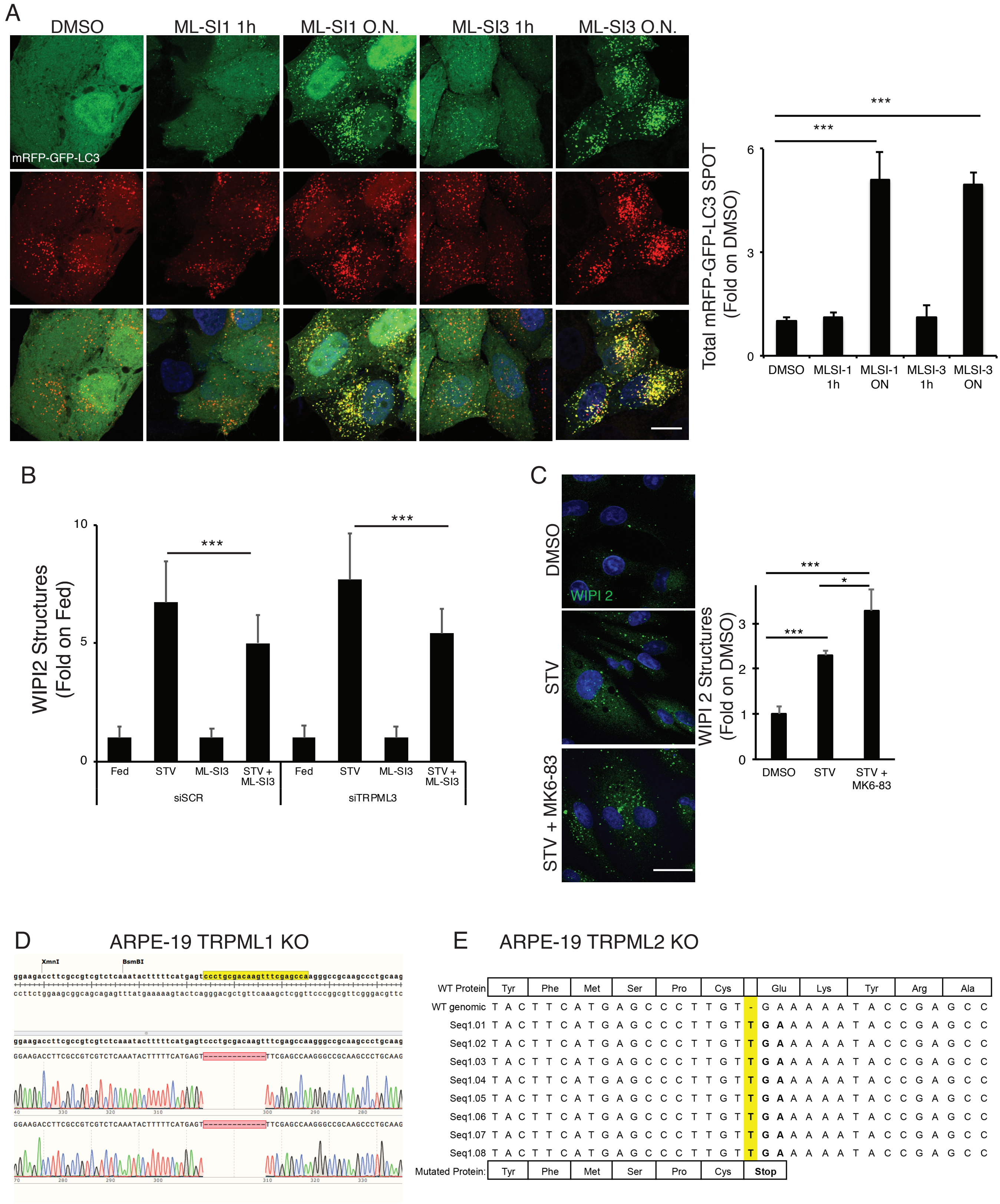

Supplementary figure 9.
(A) HeLa cells stable transfected with mRFP-GFP-LC3 were treated for 1 hour or overnight (O.N.) with ML-SI1 or ML-SI3. The plot show the total mRFP-GFP-LC3 spot per cells as fold induction to cells treated with DMSO. Values are means ± s.d. of n=720 cells pooled from 3 independent experiments. (B) Bar plot showing WIPI2 puncta quantification in ARPE-19 cells silenced with a pool of 3 siRNA oligos targeting TRPML3, upon treatment with ML-SI3. Quantification is expressed as fold induction to DMSO treated cells. Values are means ± s.d. of n>200 cells pooled from 2 independent experiments. (C) Representative confocal images of endogenous WIPI2 in ARPE-19 cells upon treatment with starvation (amino acid) +/- MK6-83. Plots show WIPI2 puncta quantification as fold induction to cells treated with DMSO. Values are means ± s.d. of n=300 cells pooled from 2 independent experiments. (D-E) Sequence analysis of ARPE-19 CrispCa9 MCOLN1 KO and ARPE-19 CrispCa9 MCOLN2 KO. P-values calculated by two tails Student's t-test. Scale bar: 20 μm. * represents p-value < 0,05; ** represents p-value < 0,01; *** represents p-value<0.001.

Supplementary Figure 10

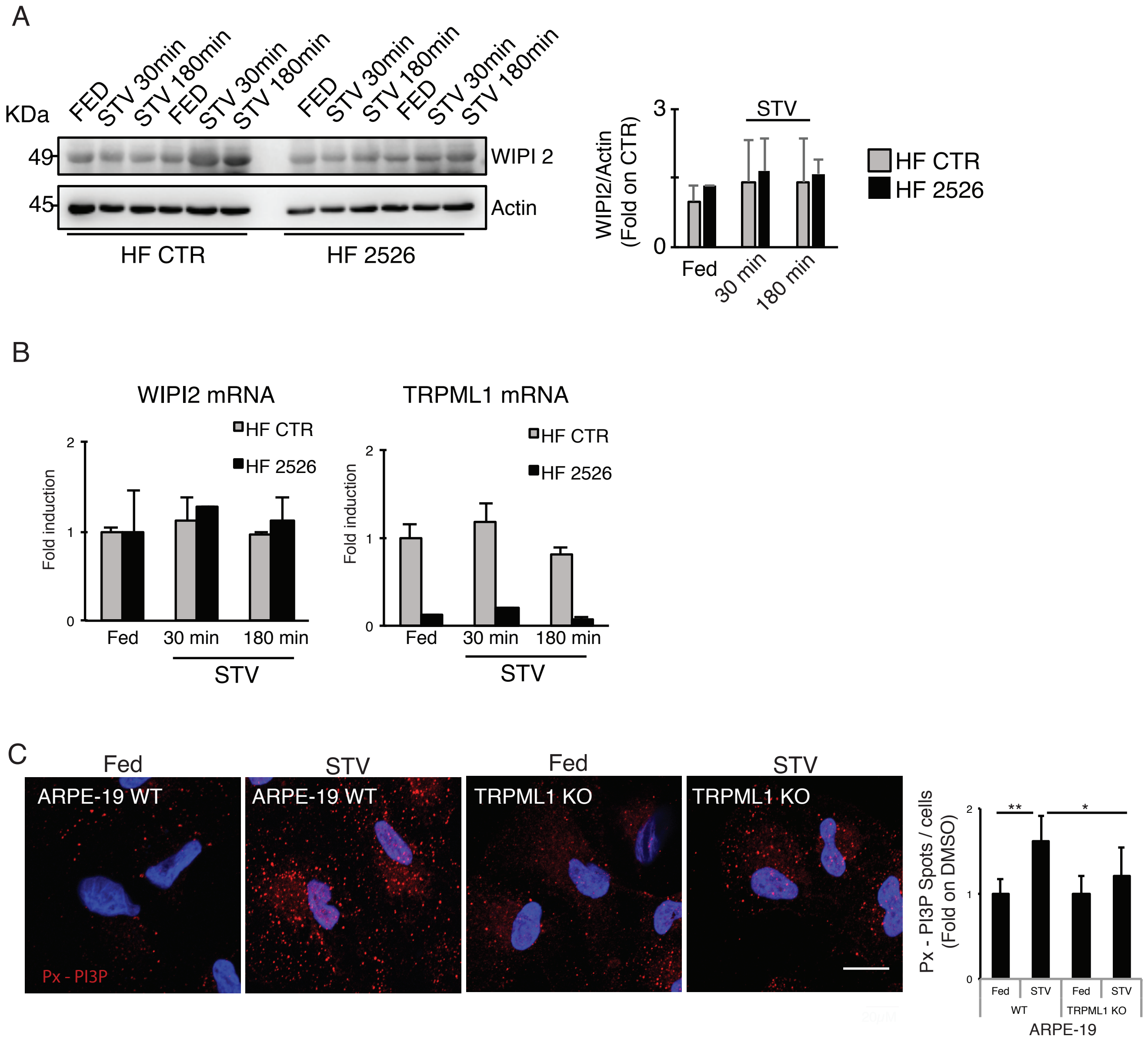

Supplementary figure 10.
(A) Representative image of immunoblot analysis of endogenous WIPI2 in human fibroblast wild type (HF CTR) and mucolipidosis type IV (HF 2526) treated with complete medium (Fed) or HBSS (30 and 180 minutes). Plot shows the densitometry of WIPI2 band normalized to Actin as fold induction to Fed treated cells. The data in the graphs on the right are mean values ± s.d., n=3 lysates per condition pooled from 3 independent experiments. (B) Quantitative PCR showing mRNA levels of WIPI2 and TRPML1 in HF CTR and HF 2526. The data in the graphs are mean values ± s.d., n=3 samples per condition. (C) Representative confocal images of PI3P puncta visualized by the PX domain of the p40phox subunit of the NADPH oxidase in ARPE-19 cells wild type or TRPML1 knock out in complete medium (fed) or starved (HBSS). The plot shows the quantification of PX positive structure per cell. Values are means ± s.d. of n=316 cells pooled from 3 independent experiments. P-values calculated by two tails Student's t-test. Scale bar: 20 μm. * represents p-value < 0,05; ** represents p-value < 0,01; *** represents p-value<0.001.

Supplementary Figure 11 - Uncropped blots

Figure 1C

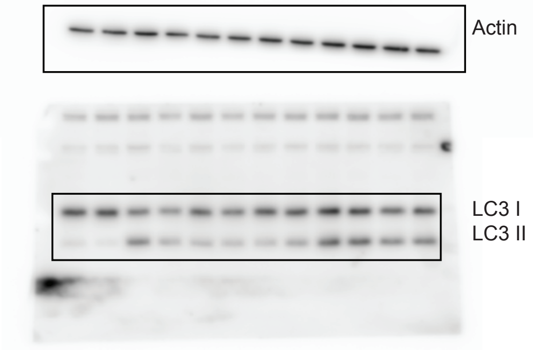

Figure 4E

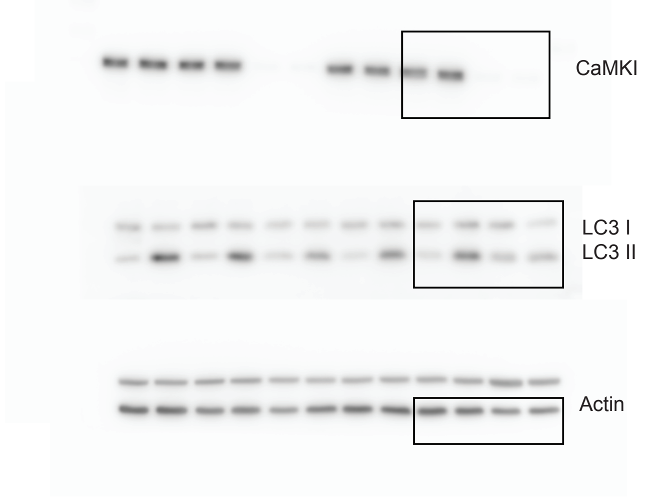

Figure 4H

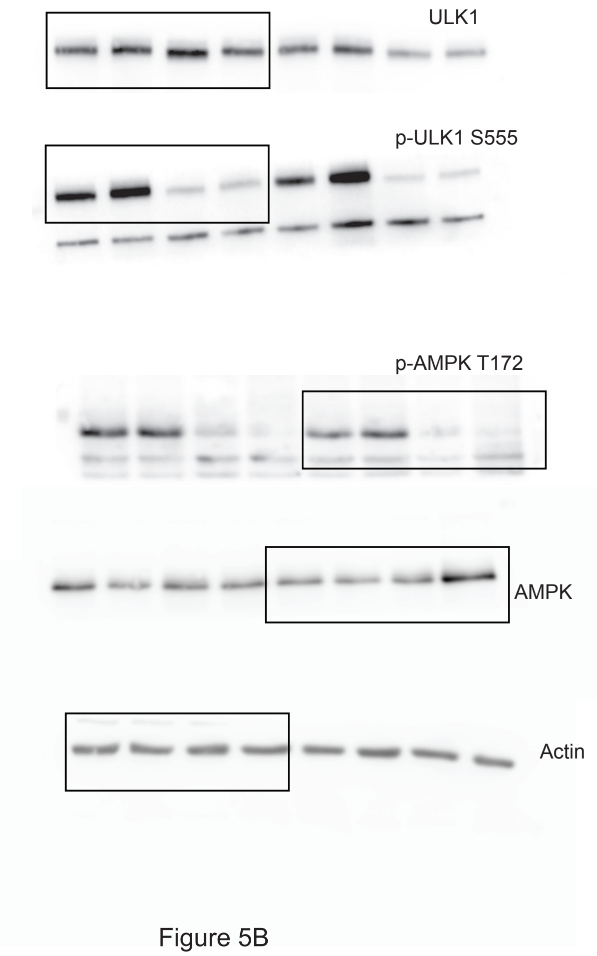

Figure 4I

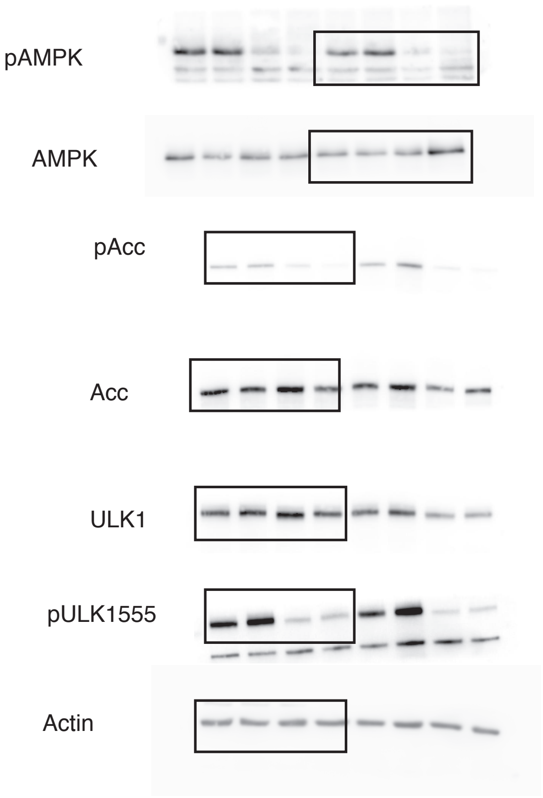

Figure 5B

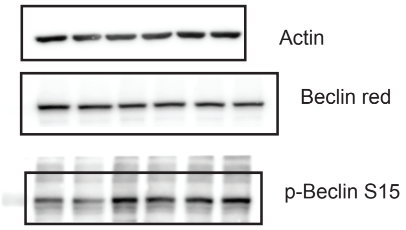

Figure 5C

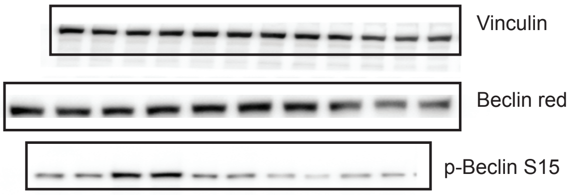

Supplementary Figure 11 - Uncropped blots

Supplementary Figure 2C

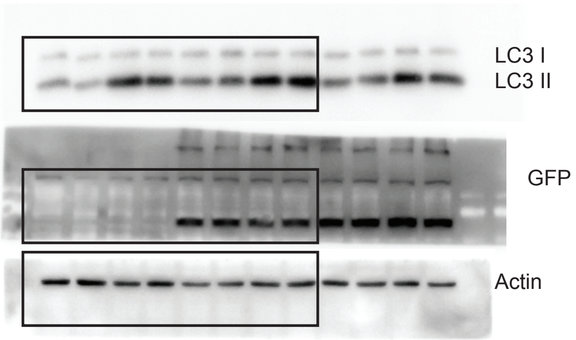

Supplementary Figure 2D

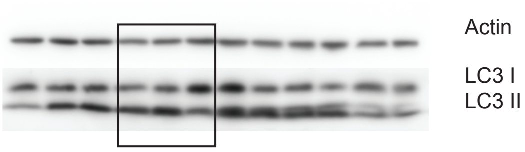

Supplementary figure 5B

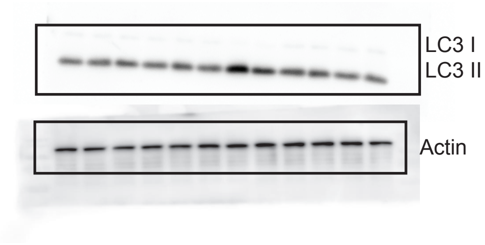

Supplementary figure 5E

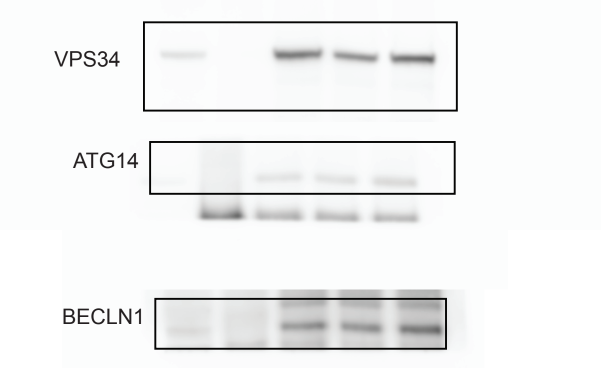

Supplementary Figure 7F

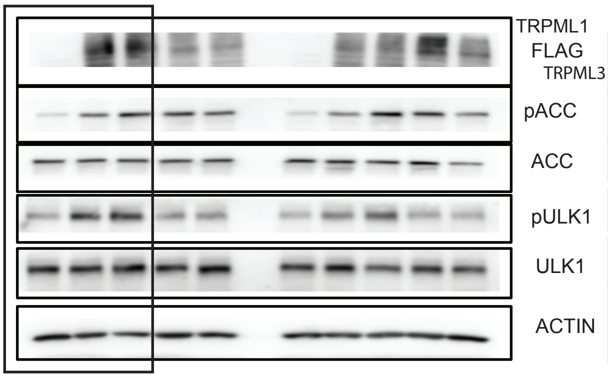

Supplementary Figure 7G

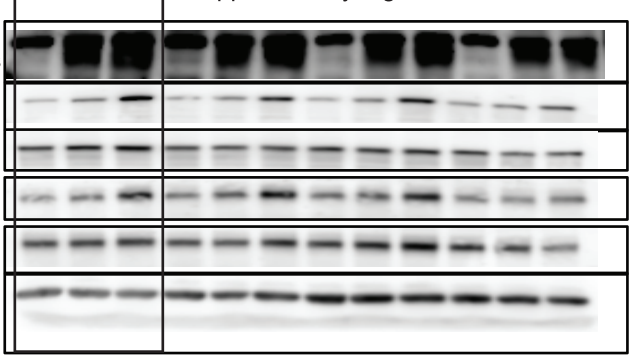

Supplementary figure 10A

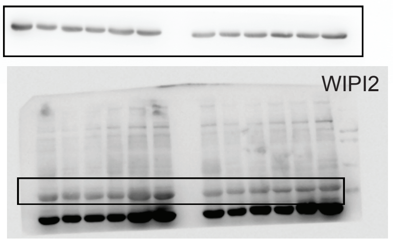

Supplement: Supplementary file 1 — Supplementary Information [file 41467_2019_13572_MOESM1_ESM.pdf]
